# Permeation of photochemically-generated gaseous chlorine dioxide on Mars as a significant factor in destroying subsurface organic compounds

**DOI:** 10.1038/s41598-024-57968-1

**Published:** 2024-04-01

**Authors:** Jacob Newmark, Samuel P. Kounaves

**Affiliations:** Department of Chemistry, Tufts University, Medford, MA USA

**Keywords:** Planetary science, Astronomy and planetary science

## Abstract

It has been shown that ultraviolet (UV) irradiation is responsible for the destruction of organic compounds on the surface of Mars. When combined with the photochemically-driven production of oxychlorines (ClO_x_) it can generate highly reactive species that can alter or destroy organic compounds. However, it has been assumed that since UV only penetrates the top few millimeters of the martian regolith, reactive ClO_x_ oxidants are only produced on the surface. Of all the oxychlorine intermediates produced, gaseous chlorine dioxide [ClO_2_(*g*)] is of particular interest, being a highly reactive gas with the ability to oxidize organic compounds. Here we report on a set of experiments under Mars ambient conditions showing the production and permeation of ClO_2_(*g*) and its reaction with alanine as a test compound. Contrary to the accepted paradigm that UV irradiation on Mars only interacts with a thin layer of surface regolith, our results show that photochemically-generated ClO_2_(*g*) can permeate below the surface, depositing ClO_x_ species (mainly Cl^−^ and ${\text{ClO}}_{3}^{ - }$) and destroying organic compounds. With varying levels of humidity and abundant chloride and oxychlorines on Mars, our findings show that permeation of ClO_2_(*g*) must be considered as a significant contributing factor in altering, fragmenting, or potentially destroying buried organic compounds on Mars.

## Introduction

Perchlorate (ClO_4_^−^) was first detected on Mars by the Wet Chemistry Lab (WCL) on the Phoenix Mars Lander at concentrations of 0.6 wt%^1,2^, by the Sample Analysis at Mars (SAM) instrument on the Curiosity Rover^3^, and subsequently in two martian meteorites^4,5^. These discoveries confirmed its planet-wide coverage and spurred questions about the role of oxychlorines (ClO_x_) in the alteration and/or fragmentation of organic molecules on the surface of Mars^6–10^.

Large quantities of organic material should be present on the martian surface from the 10^6^ kg year^−1^ infall of chondritic meteorites, comets, and interplanetary dust particles, with an assumed carbon content of over 10%^11,12^. Despite this, the Mars Viking Landers in 1976, both with gas chromatograph-mass spectrometers (GCMS) capable of detecting organic compounds at ppb concentrations, found no indigenous organics on the martian surface^13^. A variety of hypotheses were then advanced as to why none were detected^14–18^. More recently though, analyses by Curiosity’s SAM/GCMS and Perseverance’s SHERLOC have shown the presence of organics including, benzene, naphthalene, thiophenes, but most interestingly a variety of chlorinated hydrocarbons, and aromatics^19–21^. The detection of these chlorinated compounds is indicative of the role that oxychlorines play in altering organic matter at the martian surface.

A variety of processes have been hypothesized that may be responsible for the production of ClO_4_^−^ and other oxychlorines on Mars^22–26^. Photochemically however, they can be produced by ultraviolet (UV) irradiation of Cl-bearing mineral surfaces where silica (SiO_2_) and/or metal-oxides act as photocatalysts to initially generate radicals such as ^•^﻿O_2_^‒^, which can then react with Cl-bearing minerals^16,27,28^ to produce a variety of intermediate oxychlorines such as hypochlorite (ClO^−^), chlorite (ClO_2_^−^), chlorate (ClO_3_^−^) and chlorine dioxide (ClO_2_), along with ^•^OH, ^•^Cl, and ^•^OCl radicals^13,28,29^. Although physical processes (e.g., cryoturbation, wind, and impacts) could circulate regolith and expose it to UV, it has been assumed that UV irradiation only penetrates the top few microns or millimeters of the regolith to directly produce oxychlorines and degrade organic compounds^30,31^.

Of all the intermediate oxychlorines that can be produced, ClO_2_(*g*) is of particular interest. It is a highly reactive one-electron oxidant with the ability to oxidize both inorganic and organic species^32,33^. On Earth, it has been long used as an industrial bleaching and sanitizing agent, and in food/water treatment systems^34,35^. Aimed at such applications, recent studies by Jain et al.^36^ have shown that gaseous ClO_2_(*g*) can be generated by exposure of sodium chlorite (NaClO_2_) to UV irradiation and subsequent exposure to moisture. While it has not been quantified through in situ measurements on Mars, prior investigations suggest the presence of ClO_2_^−^ as an intermediate through oxidation pathways of Cl-bearing minerals before reacting to give ClO_3_^−^ and ClO_4_^−^. Conversely, ClO_2_^−^ ions, in addition to Cl^−^, ClO^−^, ClO_3_^−^, and even ClO_2_(*g*), are found to be cosmic radiolysis products of ClO_4_^−^ with exposure to γ-radiation in the context of martian soil analogs suggesting a complex matrix of evolved oxychlorine species^37–39^. Here we report an exploratory set of constrained experiments under Mars ambient conditions demonstrating the production and permeation, within a silica sand matrix, of ClO_2_(*g*) and its reaction with alanine, one of the simplest amino acids found in both chondritic and martian meteorites^40,41^. Our results show that contrary to the accepted paradigm, UV irradiation not only interacts with a thin layer at the surface, but by driving the generation of highly reactive ClO_2_(*g*), can indirectly alter or destroy organic matter below the martian surface.

### Generation of gaseous ClO_2_(***g***) and analysis of intermediate products

We conducted laboratory experiments to determine the conditions under which ClO_2_(*g*) can be generated and its ability to permeate and degrade alanine coated on silica sand-particle surfaces. The experiments were conducted in our Mars simulation chamber (MSC) (Supplementary Fig. S1), which provides a martian atmospheric composition, pressure, temperature, humidity, and Mars solar-compliant UV irradiation. We studied the reaction of the ClO_2_(*g*) by trapping the ClO_x_ products formed and analyzing them using ion chromatography (IC). In parallel, its effects on alanine were studied by identifying the resulting alteration products using GCMS.

**Figure 1:**
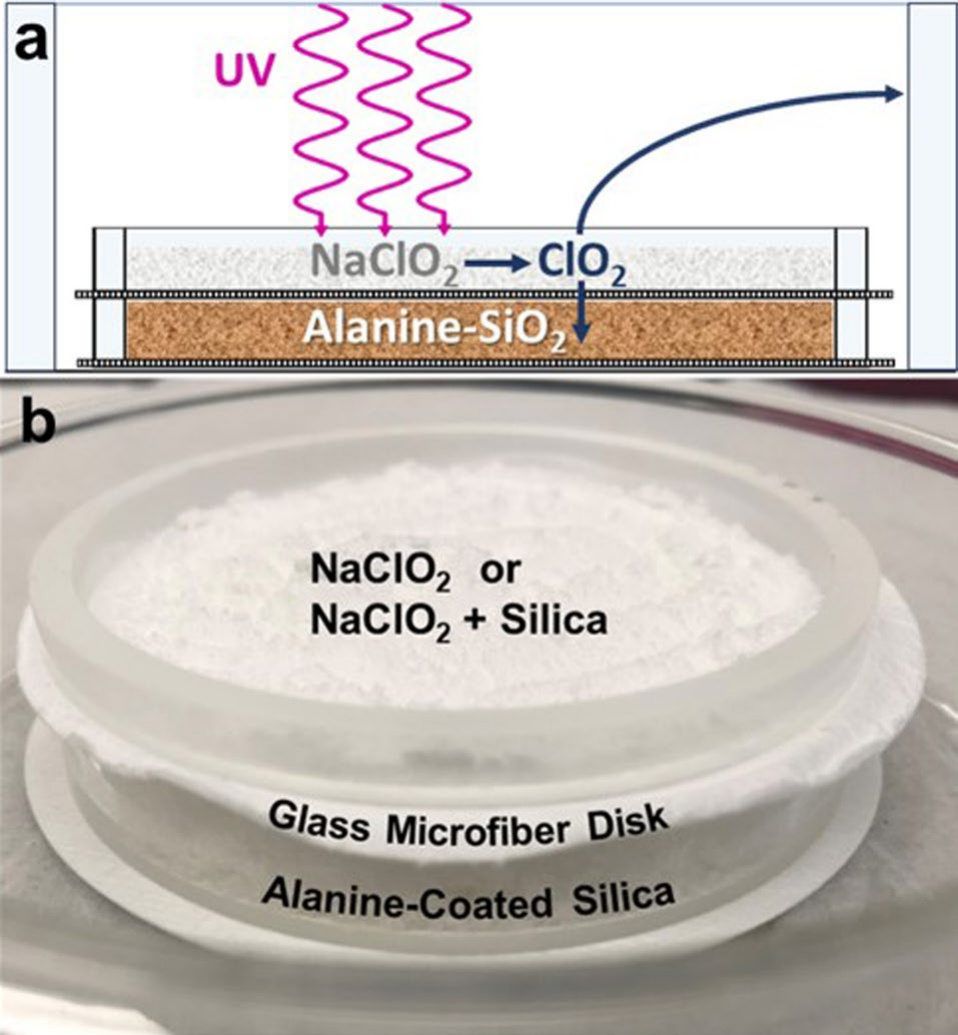
Configuration of ClO_**2**_(g) permeation chamber. (**a**) Diagram showing the outer glass cylinder surrounding two stacked inner cylinders. UV light is provided by a Xenon-arc lamp with spectrum shown in Supplementary Fig. S2. The outer cylinder provides support to keep all the components in place and also acts as a surface to prevent ClO_2_(*g*) from depositing on the stainless-steel walls of the mini-MSC. (**b**) Image of the chamber with alanine-coated sand layer separated with a 260 µm thick glass microfiber disk and stacked below NaClO_2_ or NaClO_2_ mixed with sand. Each cylinder is 5 mm in height with a 58 mm internal diameter.

By necessity, the conditions and procedures for the experiments in the MSC differed from those described by Jain et al.^36^. Instead of incremental UV activation at a specified wavelength followed by holding a relative humidity (RH) for a specific period, here to better mimic martian conditions, we continuously irradiated the NaClO_2_ layer in the permeation chamber, shown in Fig. 1, with 200–400 nm UV and with humidity constantly replenished in the MSC until a specific mass of ice-water had been depleted. Using a modified procedure reported in Jain et al., this exploratory investigation sought to better understand the implications of exposing NaClO_2_ to simulated martian UV and humidity on oxychlorine evolution and alteration of organic species. Notably, without any direct contact between the alanine and the NaClO_2_, which could react if mixed together, the ClO_2_(*g*) demonstrated permeation into the alanine-coated sand as well as diffusing to the outer glass cylinder, where on contact with the wall appears to undergo reactions leading to formation and deposition of several ClO_x_ species.

The oxychlorine products generated under different conditions by UV activation of NaClO_2_ were leached from the bottom-layer of sand, the NaClO_2_, and from any film deposited on the inner surface of the permeation chamber. In addition, to obtain information about the chemical reaction mechanisms and pathways of the generated ClO_2_(*g*), the bottom layer consisted of an alanine-coated sand. The alanine acted both as an indicator of the permeability of the ClO_2_(*g*) and provided an opportunity to investigate its ability to react with organic compounds below the UV-exposed surface.

**Figure 2:**
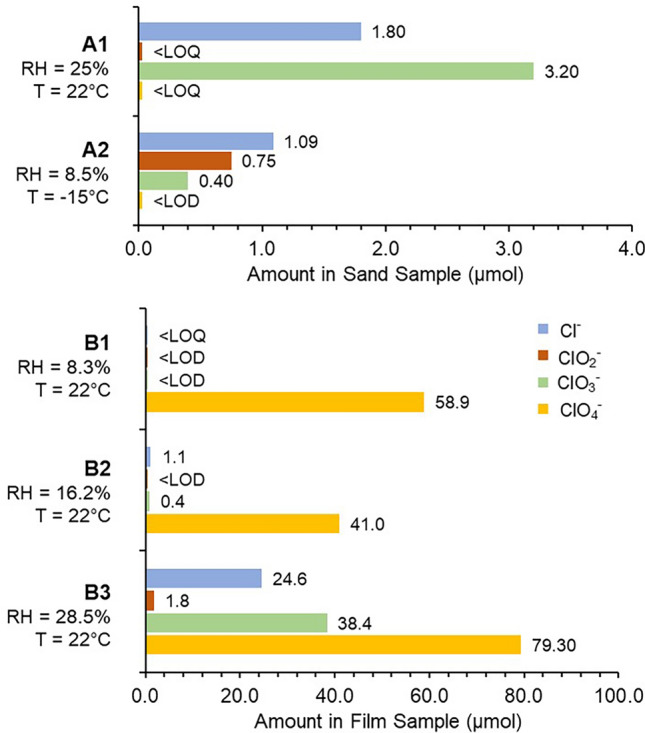
Total Amount of ClO_x_ Deposited. Bar graphs show the total amount (µmol) of each ClO_x_ deposited in the 17 g of alanine-coated sand (under conditions A1 and A2), and on the inner surface of the glass permeation chamber as a thin film residue (under conditions B1–B3), after exposure of the upper NaClO_2_ layer to UV irradiation for 24 h. Uncertainties in the µmol values and additional details are available in supplementary Table S1.* LOD* Limit of Detection, *LOQ* Limit of Quantification.

Figure 2 shows the total amount (µmol) of Cl^−^, ClO_2_^−^, ClO_3_^−^, and ClO_4_^−^ in the alanine-coated sand and the deposited thin-film at T and RH conditions (A1 and A2). Each sand sample was leached, diluted with water, concentrations of the ions determined, and the total amounts in the original sample calculated. A comparison of samples A1 and A2 shows the role of temperature and humidity in the generation and deposition of ClO_x_. In sample A1, ClO_3_^−^ was predominant, followed by Cl^−^. The amount of ClO_3_^−^ was ~ 2:1 that of Cl^−^, suggesting that following ClO_2_(*g*) generation, deposition within the sand favored the oxidized state. In sample A2, ClO_2_^−^ was present along with ClO_3_^−^ and Cl^−^, yet Cl^−^ was the most abundant, followed by ClO_2_^−^, with ClO_3_^−^ the least abundant. There appears to be a molar ratio of ~ 3:2:1 for Cl^−^:ClO_2_^−^:ClO_3_^−^, respectively, suggesting that at lower temperatures and humidity, such as on Mars, the less oxidized oxychlorines may be more prevalent as a result of the gaseous intermediate pathways. This data suggests that due to the relationship between temperature and humidity, generated radical or reactive intermediates, including ClO_2_(*g*), react with the surfaces and are converted to ClO_x_ species. As with the material selection for the MSC and the permeation chamber, sand was selected as an inert substrate (one that has been used in prior Mars simulation studies) to avoid a reaction with generated ClO_2_(*g*). This experiment was not intended to duplicate all the properties of the martian regolith and thus omits metal and mineral oxides which have the potential to react with ClO_2_(*g*). A Mars-like mineral matrix would have added further experimental and analytical complexity and may have masked any organic alternation products and ClO_x_ interaction products. Although detailed gaseous reaction data were not found for this mechanism, there are several reports of similar processes occurring in aqueous systems^36,42–44^. Due to the absorption of UV at different wavelengths for the first photodecomposition step, the reaction paths can differ depending on the wavelength^36,45,46^. It is this process that likely results in numerous stable intermediates such as Cl^−^ and ClO_3_^−^ that persisted in the MSC. A UV spectrum of the ClO_2_^−^ in solution showed peak absorptions at 210 and 260 nm as expected^47^ (Supplementary Fig. S3). This explains why numerous reaction pathways occur with higher humidity levels.

In contrast to the sand, the film from the interior wall of the outer cylinder (B1–B3) was predominantly ClO_4_^−^. At 8.3% and 16.2% RH, ClO_4_^−^ was dominant with very little or no ClO_3_^−^ and Cl^−^. At the highest RH of 28.5%, while ClO_4_^−^ was still a major ion, ClO_3_^−^ and to a lesser extent Cl^−^ comprised the rest of the film with little ClO_2_^−^. This data supports a potentially direct pathway from ClO_2_^−^ to ClO_4_^−^ with ClO_2_(*g*) as a transitory intermediate without going through ClO_3_^−^ first. With a large buildup of ClO_4_^−^ at lower humidity and lack of ClO_3_^−^, it is plausible that the ClO_4_^−^ was deposited onto the cylinder wall through reactions of ClO_2_(*g*) with •OH or other intermediates to form the ClO_4_^−^. At high RH, an equilibrium may have been reached where ClO_4_^−^ could no longer be deposited on the walls with the film containing ClO_3_^−^, Cl^−^, and ClO_2_^−^ at ratios adjusted for oxychlorine stability.

### Alteration of alanine by ClO_2_(***g***)

**Figure 3:**
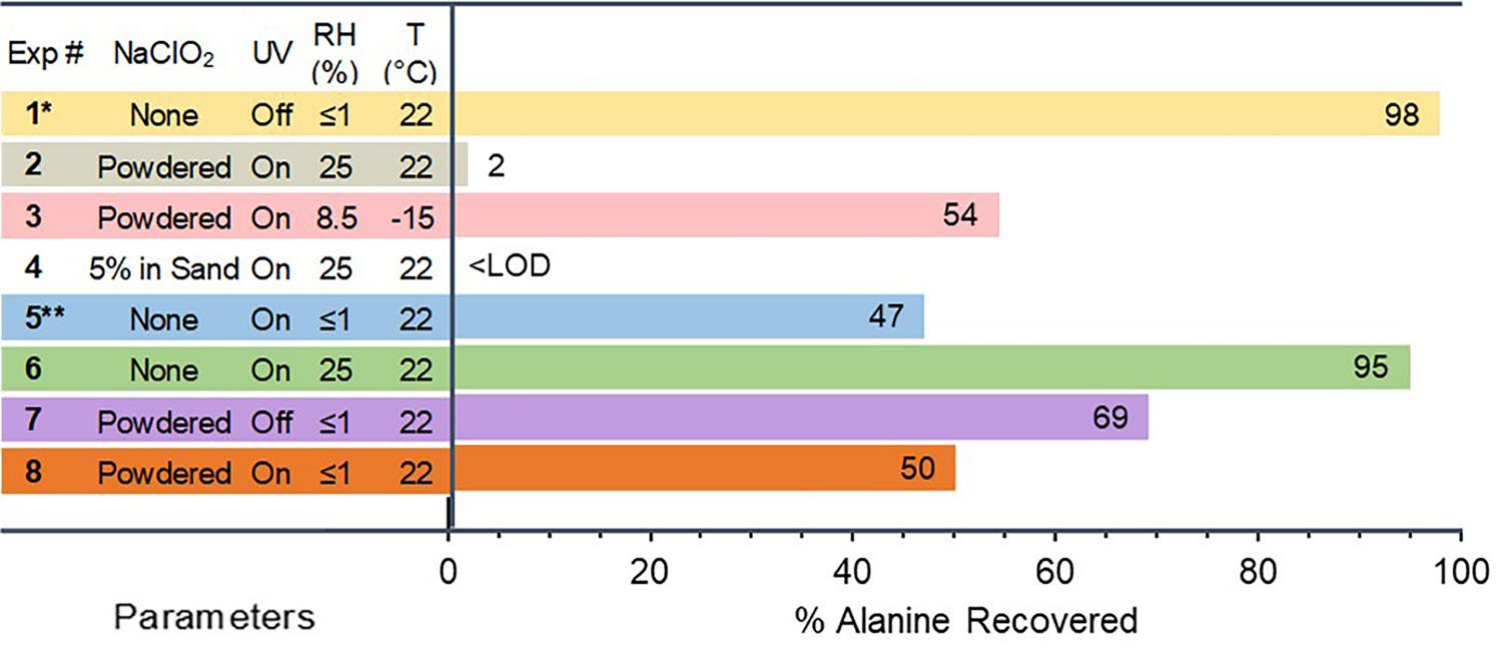
Amount of alanine remaining after each experiment. Experiments 1–4 included control samples of alanine-coated sand leachate compared with samples exposed to Mars ambient conditions, at -15 °C, with a NaClO_2_ layer, or a sand layer with 5% NaClO_2_ exposed to UV. Experiments 5–8 included UV exposure, covering alanine-coated sand, and RH in various combinations. Experiments 1–4 included duplicate or triplicate runs and their findings were averaged. All experiments were exposed to conditions in the MSC for 24 h. Uncertainties and additional details are shown in Supplementary Fig. S6 and Table S2. *Note* *Control experiment with an alanine-coated sand lower layer substrate and in a 7 mbar martian atmosphere. **Without microfiber disk covering alanine-coated sand.

The bottom layer of the alanine-coated sand (Fig. 1) was exposed to eight different conditions in the MSC, all with 7 mbar martian atmosphere. The % of alanine recovered for averaged replicates of the eight experiments, with varied conditions, is shown in Fig. 3. The control (Exp. #1) showed that when mixed with only sand and exposed to a Mars atmosphere at ~ 22 °C, 95% of the alanine was recovered, effectively 100% considering losses and error inherent in recovery and derivatization. In comparison, Exp. #2 performed under the same conditions, but with NaClO_2_ in the top layer, UV irradiation for 24 h, and ~ 25% RH, resulted in > 95% of the alanine altered to an extent that it was no longer detectable with GCMS. To better understand the reaction pathways of the ClO_2_(*g*) with the alanine, Exp. #3 repeated the procedure but with the cold plate at -15 ± 0.1 °C, thus decreasing the humidity and, in turn, the concentration of ClO_2_*(g)* following UV activation^36^, resulting in less degradation of alanine with > 50% recovery. While less effective than prior conditions, the reduced humidity and ClO_2_(*g*) generation was sufficient to degrade the alanine. After 24 h in the MSC, the RH remained at ~ 8.5%, in contrast to the experiments at ~ 22 °C where RH decreased to < 1% at that time. If the exposure in the MSC had exceeded 24 h until no humidity remained, the alanine would likely have been further altered. For Exp. #4, a sand mixture made with 5 wt% NaClO_2_ was used as the top layer instead of solely powdered NaClO_2_, a better analog of the oxychlorine availability within martian regolith, and was the most effective condition with essentially no recoverable alanine. Since the NaClO_2_ sand was made by evaporating a solution of NaClO_2_ onto the sand and then homogenizing with a mortar and pestle, the NaClO_2_ coated the sand particles such that more of it could be activated by the UV, thus generating more ClO_2_(*g*). Though not quantified, the amount of ClO_2_(*g*) thus produced was likely greater compared with powdered NaClO_2_ usage resulting in the most extensively altered alanine.

A second set of experiments sought to differentiate the generation of ClO_2_(*g*) from other variables that could individually degrade the alanine, specifically, UV irradiation, humidity, and the NaClO_2_ powdered layer. In Exp. #5, when the alanine-coated sand was exposed directly to UV irradiation, the exposed sample had a 47% recovery, suggesting that the dispersion of the alanine throughout the sand made it more susceptible to alteration directly by UV irradiation. This is consistent with previous findings where thin-films of alanine exposed to UV under martian conditions had a half-life on the order of hours^48^. This was repeated in Exp. #6 with RH of 25% at ~ 22 °C, but with the alanine-coated sand covered by a glass microfiber disk, which resulted in similar recovery as the control runs. The disk shielded the alanine from UV, independent of ClO_2_(*g*) alteration, while any water vapor dissociating to •H and •OH radicals did not impact the alanine-coated sand in subsequent recovery and analysis. In Exp’s. #7 and #8, a layer of NaClO_2_ was added to the top to analyze its impact on the recovery of alanine without and with UV exposure, respectively. Without UV exposure, ~ 70% of the alanine was recovered while with UV exposure ~ 50% was recovered. The apparent loss of alanine without UV may be related to a potential cross-contamination event in the recovery process of NaClO_2_ passing through the microfiber disk, however, this seems unlikely as a major factor of concern given the concentration of ClO_2_^−^ present in the sand leachate (Fig. 2). The sample with NaClO_2_ and UV likely interacted with other radical intermediates generated from the NaClO_2_ under dry MSC conditions. These intermediates, moisture-independent, as steps in the photo-redox process, may have impacted the recoverable alanine. There is evidence for this in the stability of ClO_2_^−^ after exposure to form Cl^−^ and ClO_3_^−^, albeit under 16.2% RH (Supplementary Table S3). Similar findings have been reported for ClO_2_^−^ alteration with samples in sand exposed to dry simulated martian conditions^49^.

### Identifying alanine alteration products

The primary goal of this study was to understand the generation and permeation of ClO_2_(*g*) on the surface of Mars and its ability to alter organics beneath the surface. A secondary goal of this work was to quantify the alanine with methodology modeled after the wet chemistry experiments of the SAM/GCMS on board the Curiosity rover that use the silylating agent N-tert-Butyldimethylsilyl-N-methyltrifluoroacetamide (MTBSTFA) to form tert-butyldimethylsilyl (TBDMS) derivatives^50^. Silylation of the carboxylic acid and amine groups of alanine, and any resulting fragments, increases the efficiency for separation by GCMS. In addition to the detection of alanine-2TBDMS being equivalent to the alanine remaining unaltered after exposure to the ClO_2_(*g*), derivatization was also successful at detecting alteration products as alanine-TBDMS and N-acetyl-alanine-TBDMS (Supplementary Fig. S4). In control samples, detection of alanine-TBDMS appeared in the range of ~ 0.3% when normalized with alanine-2TBDMS, thus below levels of significant detection, would suggest a consistent degree of incomplete derivatization of the sample. Yet after exposure to ClO_2_(*g*), the proportion of alanine-TBDMS increased significantly, particularly in the two ClO_2_(*g*) experiments at ~ 22 °C, between 10 and 30% when compared to the control sample (Supplementary Fig. S7). During derivatization, MTBSTFA was added at ~ 8 × the stoichiometric equivalent of alanine. All derivatized compounds were detected along with unreacted MTBSTFA confirmed by GCMS (Supplementary Fig. S5). A quantifiable peak of alanine-TBDMS suggests that a partial alteration of the amine may have occurred. In an aqueous solution, ClO_2_(*g*) reacts with amine groups to form chloramines^51^ and although this is a slow reaction for primary amines, during the long MSC exposure (and absence of secondary or tertiary amines) the slow rate of alteration for alanine in the solid state may explain the high levels of alanine-TBDMS. Additionally, though MTBSTFA is intended to replace the active hydrogen of polar functional groups (-OH, -NH_2_, and -SH) with TBDMS^52^, it is unlikely the same reaction would occur following chloramine alteration.

A second derivatized product, N-acetyl-alanine-TBDMS, was detected in the chromatogram. Despite the peak area vs. the alanine control, its appearance in two of three ~ 22 °C NaClO_2_ layer permeation replicates (Exp. #2) and one of each from the − 15 °C and 5% NaClO_2_ sand condition experiments (Exp. #3 and Exp. #4, respectively) confirm that it was repeatedly detected (Supplementary Fig. S8). Although the reaction mechanism for acetylation of the amine group has not been identified, the ClO_2_(*g*) may have interreacted with a fragment of another alanine molecule or the CO_2_-rich martian atmosphere. Analysis of the control alanine recovery from the sand matrix and new method development did not result in detection of an N-acetyl-alanine-TBDMS peak deriving from the stock alanine. To confirm, a commercial sample of N-acetyl-alanine was derivatized and found to have the same GCMS retention time and mass spectrum (Supplementary Fig. S5d).

The alteration products of alanine are a further indication of the complexity of the reaction, even for a simple organic compound interacting with an oxychlorine. Based on the lack of derivatization, in alanine the amine group is likely altered by ClO_2_(*g*) since both alterations identified allowed the carboxylic acid to be derivatized. Assuming that peak area is proportional to the molar concentration for similar compounds in the derivatized solution, there is a relationship between the smaller and larger peak areas of alanine-2TBDMS and alanine-TBDMS, especially in the permeation experiments at ~ 22 °C. The sum of the peak areas of the derivatization compounds (alanine-2TBDMS + alanine-TBDMS + N-acetyl-alanine-TBDMS) does not match the control alanine-2TBDMS peak area as would be expected if there were no other plausible alanine alteration products. Thus, alanine further reacted with ClO_2_(*g*) beyond chloramine alterations to compounds that could not be derivatized and detected. Based on these exploratory experimental conditions, oxychlorines on Mars would very likely react with organics via chlorination and oxidation of the original compounds following the generation of ClO_2_(*g*)^8^.

### Implications of ClO_2_(***g***) and alteration of organics on Mars

**Figure 4:**
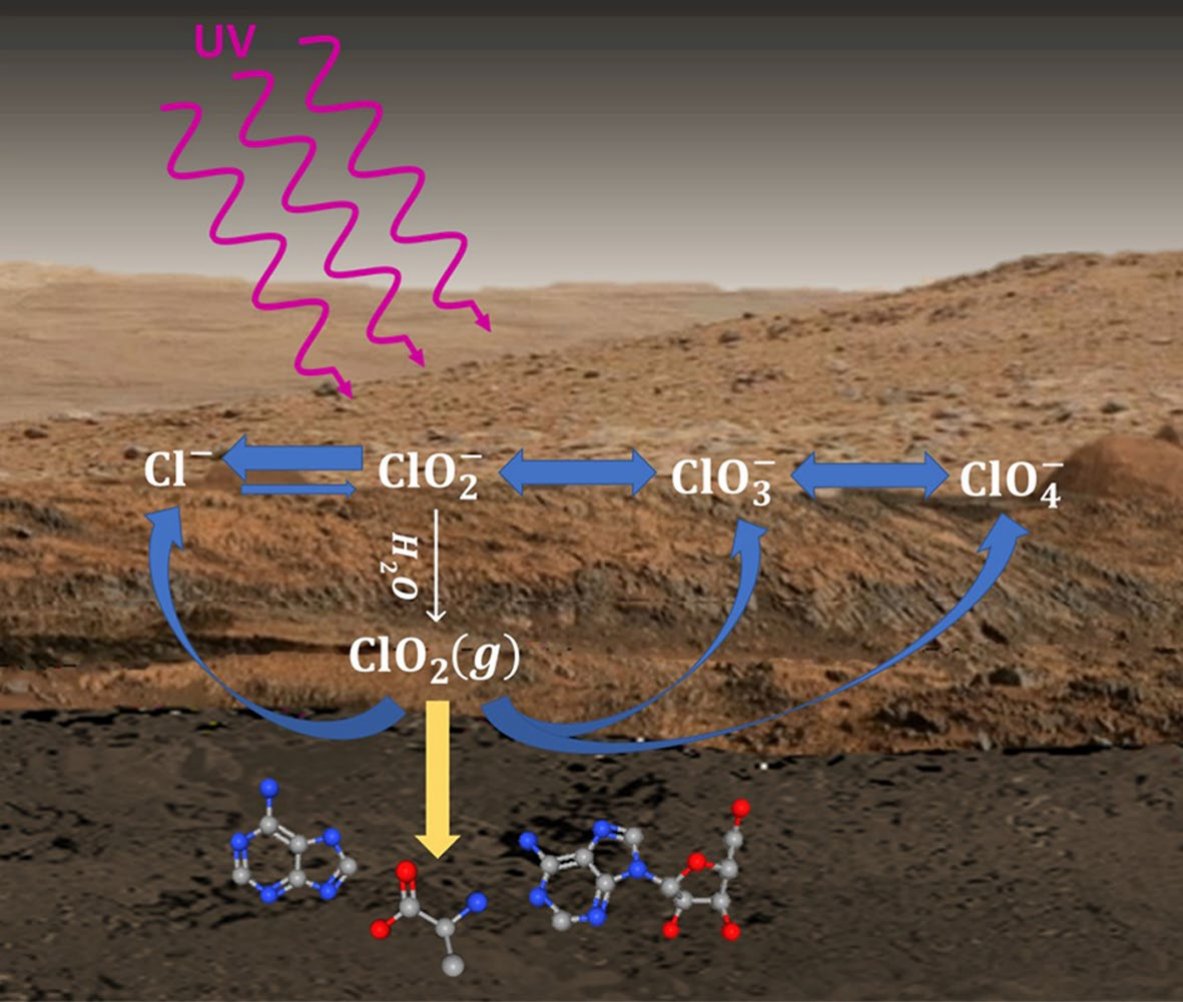
Generalized reaction pathways for generating ClO_**2**_(*g*). Diagram shows the proposed chemical reaction pathways leading to the generation of ClO_2_(*g*), during the stepwise production under Mars ambient conditions, of the oxychlorine series from Cl-bearing minerals to stable ClO_3_^−^ and ClO_4_^−^ bearing salts. Highly reactive ClO_2_(*g*) can also attack and alter organic compounds in the regolith and simultaneously can be reduced to chloride or oxidized to other oxychlorine species. *Note* the proposed reaction pathways are not balanced chemical equations.

The results of combining the ion analyses (Fig. 2) with the GCMS analyses of the alanine after exposure to ClO_2_(*g*) (Fig. 3) are shown in Fig. 4. The proposed reaction scheme takes into account both the implications of ClO_2_(*g*) permeation to alter buried organic compounds as well as the resulting deposition onto mineral surfaces after ClO_2_(*g*) generation**.** The results of this exploratory study provide new insight into the production of ClO_2_(*g*) from NaClO_2_ under Mars ambient conditions at varying levels of RH, and its implications in the degradation or destruction of organic compounds and the production of intermediate oxychlorines within the sand. The sand acts as an inert substrate with respect to ClO_2_(*g*) and thus does not hinder the alteration of buried organic compounds or the deposition of oxychlorine salts. In terms of the martian regolith containing a complex matrix of minerals and metal oxides that may also interact with ClO_2_(*g*)—this factor was not evaluated within this exploratory study given the scope of the analytical techniques. Subsequent planned experiments will investigate such properties utilizing simulant regolith samples. At ambient temperatures and humidity, alanine in the sand matrix was so extensively altered that it became nearly impossible to detect after 24 h. At lower temperatures, similar processes were observed for ClO_2_(*g*) generation but to a lesser extent due to reduced humidity and subsequent ClO_2_(*g*) resulting in increased alanine recovery. Exposure of alanine to ClO_2_(*g*) also resulted in the detection of altered compounds that were partially derivatizable and identified as alanine-TBDMS and N-acetyl-alanine-TBDMS. Permeation of ClO_2_(*g*) within the sand matrix resulted in the formation and deposition of predominantly ClO_3_^−^, followed by Cl^−^ and ClO_2_^−^, varying with temperature and humidity. A proportionally large amount of ClO_4_^−^ was also found in the film deposited on the inside of the permeation chamber cylinder. The resulting ions quantified from the sand and deposited on the interior of the permeation chamber demonstrate the relevance of the ClO_2_(*g*) evolution processes, starting from NaClO_2_, to account for the production of other oxychlorine ions. On Mars, with varying levels of humidity^53^ and abundant chloride, chlorate, and perchlorate, these findings would suggest that permeation of gaseous ClO_2_(*g*) must be considered as a significant contributing factor, among other potential processes, in the alteration, fragmentation, and total destruction of organic compounds within the immediate subsurface regolith.

## Method/procedure

### The Mars simulation chamber (MSC)

The Tufts Mars Simulation Chamber (MSC) consists of a cylindrical stainless-steel chamber with an internal diameter of 60 cm and a depth of 20 cm (~ 55 L). It is equipped with a centrally placed 25 cm diameter cold plate at the base controlled by a refrigerant circulator (SP Scientific FTS Systems, RC210C0), used to maintain the cold plate at -15 ± 0.1 °C, with the gas temperature above the cold plate ~ 0 °C. The gas composition within the MSC consists of a mixture of the major gasses present in the martian atmosphere, 95.3% CO_2_, 2.8% N_2_, 1.8% Ar, 0.10% O_2_, and < 0.1% H_2_O, delivered to the chamber at a constant flow rate of 5.00 cm^3^ min^−1^ by a mass flow controller (MKS, 1179A) with a digital power supply (MKS, PR4000). The chamber's internal pressure was maintained at 7.0 mbar for this study, monitored by an MKS type 626C Baratron® capacitance manometer controlled by an MKS 600 series pressure regulator connected to a mechanical oil-free scroll vacuum pump (Anest Iwata, model ISP 90). Refer to Supplementary Fig. S1 for the layout of the MSC and its controls. UV irradiation is delivered into the chamber by a 300 W Xenon-arc lamp (USHIO, model 6258), installed in an arc lamp housing (Newport, model 66,901), and connected to a power supply (Oriel Instrument, OPS-A500). The UV light beam was filtered to remove NIR wavelengths with a water filter (Newport, 6123NS) and limit the heating of the samples inside the chamber and then reflected 90^o^ with a dichroic beam turning mirror (Model 66,246, Newport) downwards through a fused silica port at the top of the MSC. The UV spectrum was recorded from 200 to 400 nm with a spectrophotometer probe (BLUE-Wave, StellarNet) (Supplementary Fig. S2), at a 0.1 nm resolution in n = 12 scans and then averaged. Due to the high concentration of CO_2_ in the martian atmosphere virtually no wavelengths below 200 nm reach the martian surface^54^, thus the provided range of 200–400 nm is representative of the UV impinging on the martian surface.

### Humidity and temperature control

Within the MSC, the humidity was controlled by the presence or absence of a beaker containing 25 g of ice-water, placed away from the UV beam and cold plate, at a distance of ~ 15 cm from the sample. The release of water vapor into the simulated martian atmosphere throughout the experimental duration was intended to reproduce humidity on Mars. The relative humidity (RH) within the chamber was measured every ten minutes at a 0.1% resolution (Meter, Em50 series data logger). The cold plate was used in Exp. #3 with the re-circulator set at − 15 ± 0.1 °C to analyze the effects of temperature on RH and the generation of ClO_2_(*g*). Using the cold plate, the gas temperature within the MSC was assumed to be ~ 0 °C, when not implemented, internal chamber temperatures were dictated by ambient laboratory conditions of ~ 22 °C. To begin martian simulations, the chamber was pumped down to a ~ 0.1 mbar pressure, the pressure controller was turned on to regulate the vacuum pump valve while Mars gas flowed into the chamber. Once 7.0 mbar pressure was reached inside the chamber, it was equilibrated for 30 min with constant flow and vacuum to remove any remaining laboratory gases and water vapor. When the humidity was required to be as low as possible, the ice-water beaker was removed. After equilibration, the RH was < 1.0% and considered the baseline for dry martian conditions. Mars ambient is defined as a Mars gas mixture at P = 7 mbar, T = − 15 ± 0.1 °C, and RH < 1%.

### Mini Mars simulation chamber

The MSC was fitted with a stainless-steel Mini Mars Simulation Chamber (mini-MSC) to contain the gaseous oxychlorine produced, or any other gasses, within a small volume to prevent dilution. The interior volume of the mini-MSC is ~ 700 cm^3^ with an independent sealable lid equipped with its own 10 cm diameter fused silica window nearly the width of the inner diameter to maximize sample exposure to UV radiation. The mini-MSC was placed directly on the cold plate to ensure temperature remained consistent in the mini-MSC and centered within the UV beam spot. Once sealed, the mini chamber only has a single port with tubing to the outside of the chamber to an external valve to allow for connecting the atmosphere of the mini-MSC to the larger MSC. Once equilibrated, the mini-MSC could remain connected to the MSC and receive a continuous flow of Mars gas and humidity with the expectation that generated gases would dissipate or be sealed within the chamber and retain its initial atmosphere and humidity that could not be replenished once closed. The barometer, simulant gas flow, vacuum pump outlet, ice-water beaker, and RH detector were connected to (or placed inside) the MSC, with homogeneous conditions assumed to be consistent throughout the chamber including in the mini-MSC when the valve remained open. Schematics of the mini-MSC orientation and connection with the MSC are shown in Supplementary Fig. S1.

### Permeation chamber

The permeation chamber was designed to recreate the potential dispersion of organic compounds in martian regolith with discrete layers that could be individually separated and analyzed. Inner glass cylinders were cut to have a height of 5 mm and an inner diameter of 58.25 mm to then have two cylinders stack on top of one another separated by an inert glass microfiber disk (FE Whatman®, 70 mm × 0.26 mm binder-free filter disk). The stacked inner cylinders separated by the disk were then assembled on a glass petri dish (Pyrex®). The purpose of the glass microfiber disk was to prevent any direct contact between the alanine-coated sand and NaClO_2_ which could react upon mixing. Once complete, the outer glass cylinder with a height of 76.4 mm and internal diameter of 66.5 mm was placed around the stacked cylinders to prevent disturbance of the inner cylinders (Fig. 1). Once the sample materials had been prepared and assembled in the permeation chamber, it was placed in the mini-MSC and sealed within the MSC. Following atmospheric equilibration with simulant gas, samples were exposed to MSC conditions for 24 h in the presence or absence of UV irradiation, humidity, and temperatures for desired experimental conditions.

### Sample preparation

Sand (SiO_2_, Supelco, 40–100 mesh) was washed five times with Nanopure™ 18.2 MΩ-cm deionized (DI) water (Thermo Scientific, Barnstead™) to remove fine particles and ion contaminants, then dried overnight in a gravity convection oven (Quincy Labs, Model 10) at ~ 80 °C. Alanine (Sigma-Aldrich, BioXtra® ≥ 98.5%, homochiral L-alanine) was weighed to the nearest 0.1 mg (Mettler Toledo Balance, XS64) and mixed in DI water to a stock solution concentration of 1,000 ppm, then sonicated at 40 kHz (Branson Model-1510) for one minute to ensure all materials had been dissolved and then stored at ~ 4 °C. The chirality of amino acids in D and L-form does not play a role in UV photolysis^55^ and is thus referred to as alanine. The sand coated with alanine (alanine-coated sand) mixture was prepared by mixing ratios of 10.00 g sand, 2.0 mL of 1,000 ppm alanine stock solution, and 1.0 mL DI water and oven drying the liquid mixture at ~ 60 °C overnight to avoid degradation of the alanine. Once dried the alanine-coated sand was homogenized using a mortar and pestle. For each experiment, two replicates of 17.000 ± 0.005 g alanine-coated sand were measured—one was covered and placed in a drawer as a control sample while the other was used to fill the bottom inner cylinder of the permeation chamber.

Sodium chlorite (NaClO_2_, Sigma-Aldrich, technical grade 80%) was ground with a mortar and pestle to a fine grain size, then 1.000 ± 0.005 g was weighed and placed in the upper inner cylinder of the permeation chamber. The NaClO_2_ was evenly distributed to cover the entire surface area of the cylinder separated from alanine-coated sand by a glass microfiber disk. To prepare the 5% by weight NaClO_2_ sand (5% NaClO_2_ sand) utilized in Exp. #4, 2.0 g of NaClO_2_ salt was dissolved in 12.0 mL of DI water. This solution was then mixed with 38.0 g of cleaned sand and dried overnight at ~ 60 °C. When dry, the 5% NaClO_2_ sand was homogenized with a mortar and pestle and 17.000 ± 0.005 g was weighed and filled the top level of the permeation chamber above the alanine-coated sand layer.

### Sample recovery and derivatization

Following exposure in the MSC and disassembly of the permeation chamber, alanine-coated sand was transferred to a 20 mL borosilicate glass scintillation vial (Fisherbrand™) and mixed with 8.0 mL of DI water to maximize the leaching of amino acid from the sand alongside the identical control sample of alanine-coated sand set aside before MSC exposure. Both scintillation vials were mixed with a magnetic stir bar (Fisherbrand™, 12.7 mm × 3.2 mm PTFE) simultaneously at 750 rpm (Cimarec i Poly 15) for one hour. Each leachate was transferred to a 5 mL syringe (BD, Luer-Lok™ Tip) and filtered through a 0.2 µm filter (Nalgene™ sterile filter) into borosilicate glass Dram vials (Fisherbrand™) and sealed.

Alanine samples were prepared for derivatizing with N-tert-Butyldimethylsilyl-N-methyltrifluoroacetamide (MTBSTFA, Supelco, ≥ 99.0%) (Supplementary Fig. S12) by transferring 588 µL of the recovered alanine leachate into a 2 mL glass autosampler vial (Agilent, screw cap PTFE silicone septa). The solution was then evaporated uncapped in an oven overnight at ~ 65 °C. A thin film was observed in the dried vials to which 190 µL of acetonitrile (Fisher Chemical, HPLC grade) was added followed by 10 µL of the MTBSTFA. The mixture was capped and sonicated for one minute then heated to ~ 65 °C for one hour to maximize derivatization reactions. Once cooled, the mixture was diluted with 800 µL of acetonitrile such that the derivatized alanine would have a potential concentration of 250 ppm. This method has been tested extensively in the context of martian amino acid detection and parallels the capabilities and methods of the SAM instrument suite^56–58^. This technique is especially useful in the context of oxychlorine ions, specifically ClO_4_^−^ which does not impede or influence the derivatization of organic compounds injected for GCMS analysis^59^.

### Gas chromatography-mass spectrometry (GCMS) methods and analysis

Separation and analysis of organic samples was performed using an Agilent 6890N gas chromatograph equipped with a 7683 automated sampler and connected to an Agilent 5973N mass selective detector (MSD). The GC inlet was used in split mode at a ratio of 20:1 and set to a temperature of 260 °C with 1 µL of sample injected for analysis. Separation was performed using a 30 m × 0.320 mm × 0.25 μm low polarity Agilent J&W, HP-5 ms ultra inert capillary column. Helium was used as a carrier gas at a flow rate of 1.0 mL min^−1^. The initial column temperature was set to 160 °C, then after one minute, the oven was heated at a rate of 10 °C min^−1^ until it reached 200 °C and maintained there for one minute. The MS solvent delay was set for 1.20 min to avoid large solvent peaks from the acetonitrile. The MSD was set to a scan range of 40–550 m*/z*, an electron ionization energy of 70 eV, the MSD transfer line at 280 °C, the MSD Source at 230 °C, and the MSD Quad at 150 °C. GC peaks were identified using the Agilent ChemStation software loaded with the NIST20 MS library.

Once samples were exposed to martian conditions, they were leached, derivatized, and analyzed on the GCMS as rapidly as possible to limit any further degradation. Each experiment’s control and MSC-exposed samples of alanine-coated sand were compared against one another and not directly in sequence with samples from other experimental conditions. To ensure consistency and avoid systematic errors, control and exposed samples were injected in triplicate in a random order with acetonitrile blank samples between each pairing to confirm that no residual sample remained in the GCMS. Integrated peak areas of the triplicate injections for the control and exposed samples were averaged and the exposed sample was normalized to the control.

### Ion chromatography methods and analysis

In addition to the analysis of alanine following MSC exposure, the ionic species in the sample were analyzed using Ion Chromatography (IC). The leachate solution of extracted alanine was also analyzed for ClO_x_ within the alanine-coated sand to compare control and exposed samples. Following sample exposure in the MSC, a thin film was observed to build up on the inner walls of the outer glass cylinder beginning above the NaClO_2_ layer (Supplementary Fig. S11). To analyze this material, a sterile cotton swab (Curity) was wetted in a Dram vial containing 2 mL of DI water and vigorously swirled around the interior of the cylinder to dissolve the film residue then dipped back into the Dram vial to transfer the dissolved ions. This process was repeated five times to maximize the recovery then filtered through a 0.2 µm filter to remove solid particulates. The objective of this method was to minimize the dilution of the recovered film sample since the residue could not be fully recovered. Before assembly of the permeation chamber, the outer cylinder was thoroughly cleaned with DI water and a laboratory detergent (Alconox, Precision Cleaner) then heated to ~ 65 °C to dry the surface. The cleaned cylinder was swabbed with the same method and considered the control to quantify any background oxychlorine material present before exposure.

IC analysis (Dionex Aquion) was used to quantify the concentrations of chloride, chlorite, chlorate, and perchlorate with a dynamically regenerated anion suppressor (DRS 600). To analyze ClO_4_^−^, samples were injected from a 100 µL sample loop onto a Dionex 4 mm × 250 mm Ionpac AS16 analytical column, after passing through a 4 mm × 50 mm AG16 guard column. The 35 mM potassium hydroxide eluant was diluted from a stock solution (KOH, Alfa Aesar 50% w/v solution) with a flow rate of 1.00 mL min^−1^ and a suppression current of 87 mA. For the analysis of Cl^−^, ClO_2_^−^, and ClO_3_^−^, a 25 µL sample loop was used along with a Dionex 4 mm × 250 mm Ionpac AS18 analytical column, and a 4 mm × 50 mm AG18 guard column. A 23 mM KOH eluant was prepared and passed through the system at a flow rate of 1.00 mL min^−1^ with a suppression current of 57 mA.

Before sample analysis, standard samples were analyzed to calibrate the sequence and identify peaks based on retention time (Supplementary Fig. S10). For ClO_4_^−^, standards were diluted from a stock solution (SPEX CertiPrep, 1000 ppm) in DI water with quantification between 156 and 20,000 ppb. A matrix standard was prepared from anion stock solution for Cl^−^ (Dionex, 30 ppm), ClO_2_^−^ (Inorganic Ventures, 1,002 ppm), and ClO_3_^−^ (Honeywell Fluka, 1,000 ppm) with quantification between 187 and 3,000 ppb, 156–5,010 ppb, and 156–10,000 ppb, respectively. Sample leachate and recovered film solutions were diluted with DI water to 1:10 and 1:100 to ensure quantification at high concentrations. Following the standard curve samples, diluted solutions were injected in triplicate to ensure consistency in quantification with a DI water blank between sets of three.

### Analysis of the sodium chlorite after exposure

Following the film generation and recovery experiments, a sample of the NaClO_2_ layer from the 16.2% RH experiment was dissolved in DI water at a 1,000 ppm concentration alongside a control sample of NaClO_2_ dissolved to a 1,000 ppm solution that had not been exposed to simulated martian conditions. Results of sample analysis for Cl^−^, ClO_2_^−^, ClO_3_^−^, and ClO_4_^−^ are presented in Supplementary Table S3. There was limited detection of ClO_3_^−^ and no detected peaks for ClO_4_^−^ in the control sample suggesting that the 80% technical grade commercial NaClO_2_ does not contain further oxidized oxychlorine ions. Following exposure to the MSC with UV irradiation and isolated within the mini-MSC at 16.2% RH, the concentration of ClO_2_^−^ decreased by a factor of two while the concentration of Cl^−^ increased. Additionally, ClO_3_^−^ was detectable with a nearly tenfold increase in abundance. The increase in Cl^−^ along with the appearance of ClO_3_^−^ suggests that under humid martian conditions, the lifespan of chlorite salts may be limited following formation as it rapidly evolves through photochemical redox pathways.

### IC Analysis for clarifying role of T and RH in the generation and deposition of ClO_x_

IC analysis of the sand leachate solution from experiments at ~ 22 °C and − 15 ± 0.1 °C (Table S1) clarifies the role of temperature and RH in the generation and deposition of ClO_x_. At ambient temperatures (A1), ClO_3_^−^ was the predominant species in the leachate followed by Cl^−^. Both ClO_2_^−^ and ClO_4_^−^ were detected but were < limit of quantification (< LOQ). Conversely with the sample leached from alanine-coated sand in the MSC at − 15 ± 0.1 °C (A2), a significant decrease in ClO_3_^−^ was measured along with reduced Cl^−^ while ClO_2_^−^ was quantifiable and detected at a greater molar ratio. In comparing the two leachates, it could be assumed that environmental RH dictates the tendency for deposited species to be further oxidized to ClO_3_^−^ and potentially ClO_4_^−^ at higher RH or reduced to ClO_2_^−^ and Cl^−^. Temperature may play a role in this pathway either from a kinetic standpoint of available thermal energy or more simply as a function to dictate the RH levels. It is important to note that the leachate experiments are specifically to consider the oxychlorine presence as a result of the alteration of buried organic compounds like alanine, further experiments could be repeated without the presence of any added organics to the sand material and understand how ClO_2_(*g*) is deposited within the surface regolith. It is also possible that the ambient temperature experiment’s concentration of ClO_3_^−^ had to do with the alteration of detectable alanine thus allowing the buildup of ClO_3_^−^ oxidizing agent without any organics to further oxidize. As alanine was detected in the − 15 ± 0.1 °C permeation experiments to a greater extent, the ClO_2_^−^ and Cl^−^ may have been the result of ongoing oxidation with further amino acid remaining to alter at the time conclusion of the 24 h experiment.

The difference in buildup of ClO_3_^−^ concentration in the leachates while ClO_4_^−^ built up as the dominant component of the film matrix may be due in part to the gas flow and vacuum pump systems implemented in the MSC. In the leachate experiments, the mini-MSC remained interconnected with the atmosphere of the larger MSC to get a continuous and measured RH, potentially at the loss of generated gaseous intermediates before depositing on the outer cylinder walls or within the sand. For the film recovery experiments, the mini-MSC reached a target RH and then was sealed off from the larger MSC chamber to minimize any loss of gaseous and oxychlorine materials. In measuring both the upward movement of ClO_2_(*g*) as well as its downward permeation into the sand, this investigation contextualized the mobility of the gaseous intermediates generated from NaClO_2_ exposure to UV irradiation and RH under martian conditions.

### Analysis of film residue

During preliminary experiments in the MSC, when NaClO_2_ was exposed to UV and humidity, a thin opaque film was deposited on the inner walls of the outer cylinder of the permeation chamber (Supplementary Fig. S11). To isolate the reaction and deposition of ClO_x_, a layer of NaClO_2_ was prepared without the alanine-coated sand layer beneath it to isolate the gaseous products from interaction with sand or alanine. After attaining Mars ambient conditions in the MSC with an ice-water beaker, the chamber RH in the outer chamber was closely monitored, and once a target RH was reached the mini-MSC was sealed off from the MSC. By avoiding the constant flow of Mars gas and the pull of the vacuum pump in MSC experiments, it is assumed that the stagnant environment of the isolated mini-MSC allowed for the slow consumption of humidity and continuous production of ClO_2_(*g*) for the 24 hour experimental duration.

As the chamber RH could not be directly controlled, IC analysis of swab-recovered films (Table S1) was the best way to quantify the Cl^−^, ClO_2_^−^, ClO_3_^−^, and ClO_4_^−^ produced as a function of RH. Before chamber exposure, the outer cylinder walls were swabbed as a control sample. Cl^−^ was detected but at a concentration < LOQ. This may have been a result of the cleaning process for the glass cylinder or trace amounts of Cl^−^ in the DI water or Dram vial, but at levels below what would have a direct impact on the recovered film.

The mini-MSC was isolated within the larger chamber, at the time of valve closing, RH was recorded as 8.3%, 16.2%, and 28.5% for the three experiments at ambient temperatures of ~ 22 °C. While Cl^−^ was detected in all exposure conditions and dilutions, the peaks were < LOQ at 8.3% RH, while at 16.2% RH had a quantifiable concentration of Cl^−^, yet this was significantly less than the detected concentration of the 28.5% RH film. ClO_2_^−^ was detected only in the film from 28.5% RH with quantification at comparatively low concentrations to the other ionic species. The ClO_3_^−^ was quantified only for the 16.2% and 28.5% RH, with the 28.5% RH film having ~ 100 × soluble ClO_3_^−^ recovered compared to the 16.2% film. The most significant ionic trend in this analysis surrounded the abundant detection of ClO_4_^−^, where at each RH level ClO_4_^−^ was at the highest concentration of the ionic species and thus considered to be the bulk of the film material. The 28.5% film had nearly twice the concentration of ClO_4_^−^ than the 16.2% sample while the 8.3% detected ClO_4_^−^ between the two. While the concentration of ClO_4_^−^ is anticipated to increase with RH, this lower detection at 16.2% RH may be due to the limitations of the film recovery method through swabbing of the interior walls of the outer cylinder that the entirety of the material may not have been recovered.

### Procedure for alanine recovery

Alanine-coated sand was exposed to various control and exposure conditions in the MSC to analyze the potential for recovery of alanine-2TBDMS through derivatization with MTBSTFA. Samples were prepared and then exposed to experimental conditions for 24 h during which time the flow of Mars gas remained constant along with the chamber pressure. Figure 3 and Supplementary Fig. S6 show the impact of UV exposure and RH at both ambient temperature and with the cold plate set to − 15 ± 0.1 °C. For each experiment, a control sample of alanine-coated sand was prepared in parallel with the exposed sample, then both samples were recovered, derivatized, and quantified with GCMS simultaneously. Each sample was derivatized in a single vial and then injected three times as technical replicates to ensure consistency in instrumental separation and peak integration. The peak area of these injections was normalized against the injection with the greatest area to a percentage value recovered such that each MSC experiment could be directly compared with one another. Peak area is a function of alanine-2TBDMS concentration in the vial, however, the sensitivity of the derivatization step to laboratory temperature and water vapor can impact the quantification and is not intended as an experimental variable. Derivatization of amino acids has been shown to have a high yield of reactivity at a rate of 90–100% of available molecules in a pure solution^56^. It should be assumed that approximately the same degree of material was quantified on GCMS but not to the extent seen by direct quantification of the molecules with other analytical techniques, seeing that GCMS has a ‘side effect’ of destroying rather than detecting labile molecules.

The MSC control experiments confirmed that the Mars ambient conditions did not have an impact on the ability to recover or derivatize alanine from the sand material. Once exposed to water vapor from the ice-water beaker in the MSC at ~ 22 °C, the RH remained constant at ~ 25% RH before declining after 8–10 h and reaching a similar RH to control dry experiments after ~ 12 h (Supplementary Fig. S9) when all the ice-water had evaporated. These experimental conditions, repeated in triplicate, had a normalized alanine-2TBDMS recovery average of 2%, from exposed alanine-coated sand compared to control samples with > 98% normalized recovery. When the cold plate was utilized at − 15 °C, a decrease in relative humidity was seen to ~ 8.5%, however with less ice-water vaporizing the RH remained consistent throughout the 24-h experimental duration. With reduced temperature and RH, an average 54% of alanine-2TBDMS was detected compared with 97% normalized peak area in duplicate experiments. A third set of experiments sought to analyze the effects of NaClO_2_ dispersed throughout sand separated by the glass microfiber disk barrier. With a 5% NaClO_2_ sand layer directly above the alanine-coated sand layer, not only would the surface area of NaClO_2_ exposed to UV irradiation decrease but the inner cylinder would be ~ 10 mm in height of combined sand instead of 5 mm of alanine-coated sand with 1 g of NaClO_2_ layer atop it. Alanine was not detected at the retention time in either replicate compared to control samples with an average 96% normalized peak area. This suggests that despite the depth of alanine material increasing, the mixture of NaClO_2_ within the sand has the greatest potency for alanine alteration. Results of these experiments are shown in Table S2.

### Supplementary Information

Supplementary Information.

## Data Availability

All numerical data in this paper are provided in the figures and the Supplementary Information in tabular form.
