# Supplementary material for: Permeation of photochemically-generated gaseous chlorine dioxide on Mars as a significant factor in destroying subsurface organic compounds
Source: Sci Rep. 2024 Apr 1;14:7682. doi: 10.1038/s41598-024-57968-1 (PMC10985076; doi:10.1038/s41598-024-57968-1)
Supplement: Supplementary file 1 — Supplementary Information. [file 41598_2024_57968_MOESM1_ESM.pdf]

Supplementary Material¶
¶
¶
¶
¶
Article

Permeation of photochemically-generated gaseous chlorine dioxide on Mars as a significant factor in destroying subsurface organic compounds¶

¶
¶
In the format provided by
the authors and unedited¶
¶

Jacob Newmark and Samuel P. Kounaves

Department of Chemistry, Tufts University, Medford, Massachusetts, USA

This document includes:

Supplementary Figures S1 to S12

Supplementary Tables S1 to S3

Supplementary Figures S1-S12

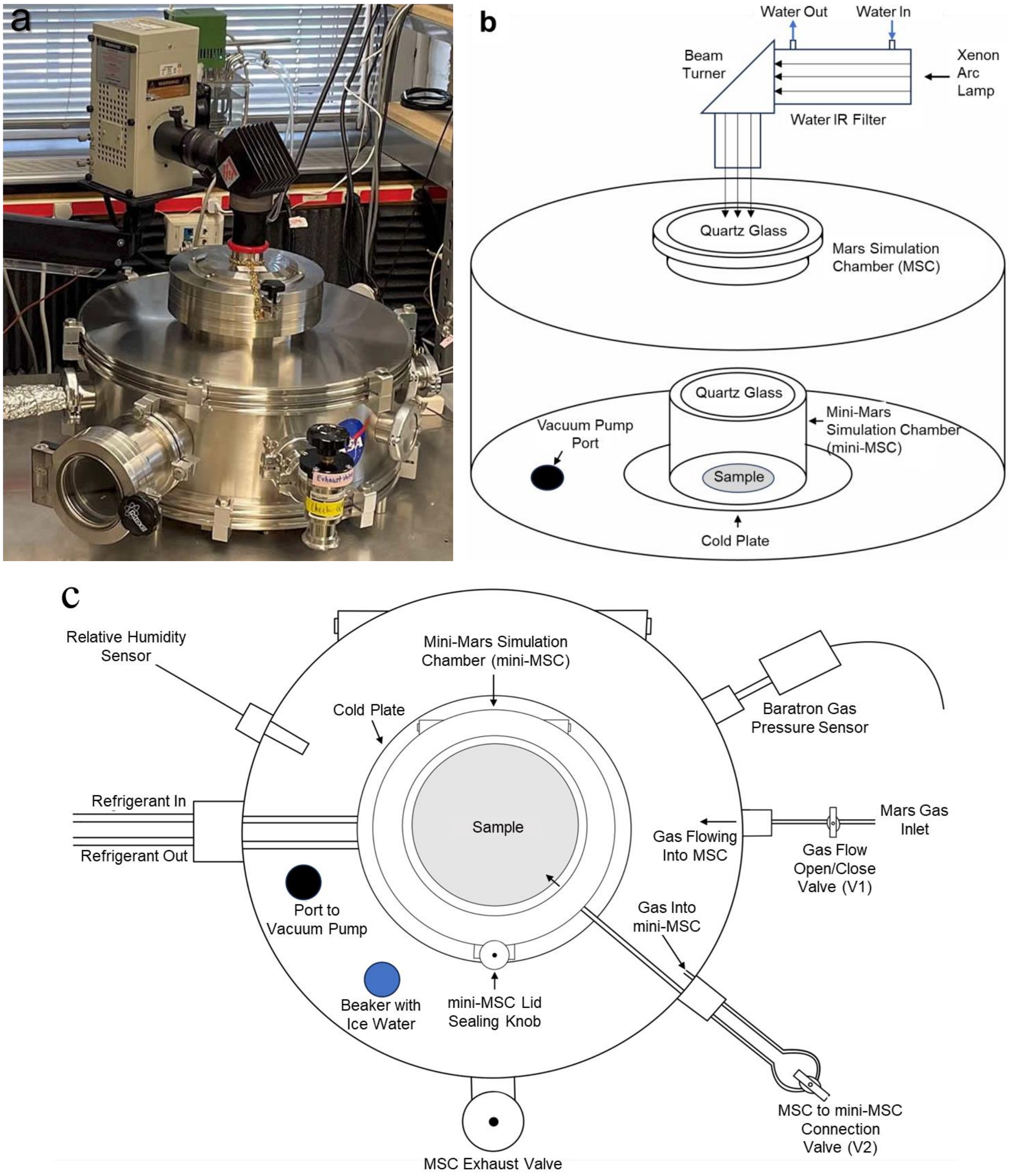

Supplementary Figure S1 | MSC and mini-MSC utilized for experimental simulation of the environmental conditions of Mars. a, The MSC and connected instruments. **b,** Side view of the MSC showing 3D aspects of the mini-MSC in the chamber and the UV light path. **c,** MSC top view showing components located within the MSC and their connection to external instruments without the chamber top cover. Tubing connecting the atmosphere of the MSC to the mini-MSC can be opened or sealed with V2.

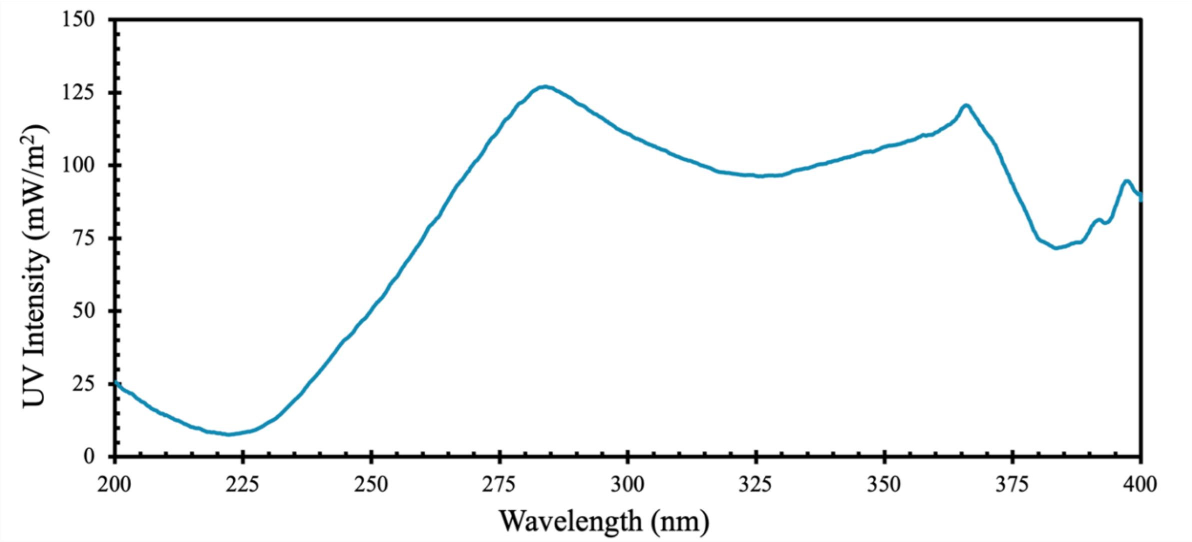

Supplementary Figure S2 | The spectrum of the UV delivered in the MSC. The UV spectrum produced by the 300 W xenon-arc lamp passed through an NIR water filter to remove wavelengths >950 nm. The spectrum was recorded using a UV spectrophotometer probe (BLUE-Wave, StellarNet) from 200-400 nm with an instrumental sensitivity of 0.1 nm. The spectrum intensity data provided is the average intensity at every tenth of a nm from n = 12 complete recorded spectrums. Following the recording of this spectral data, the lamp spectrum is assumed to have remained consistent through the MSC experiments.

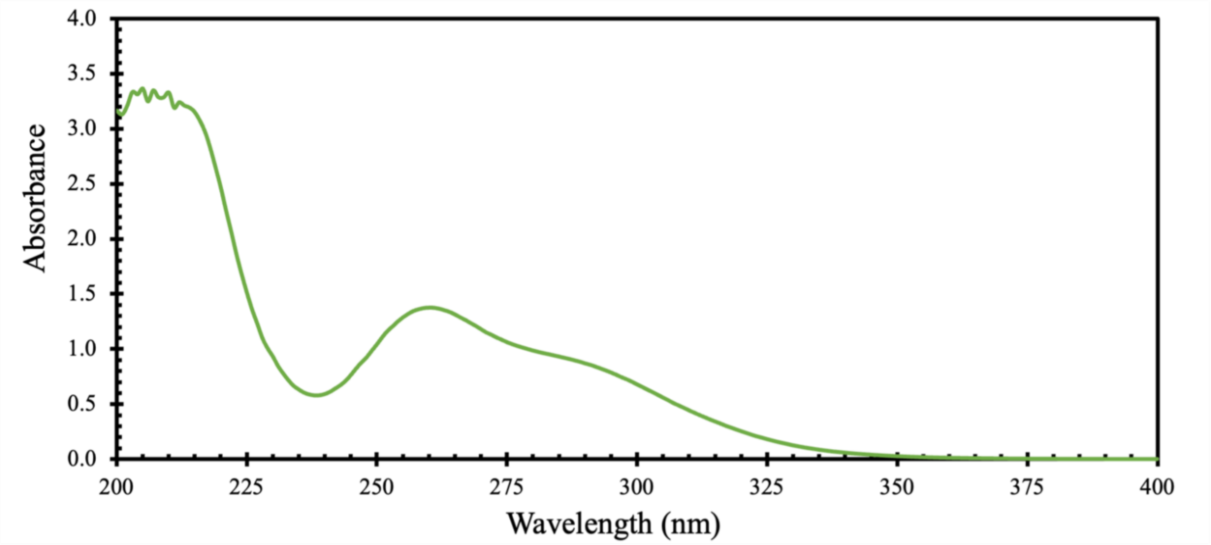

Supplementary Figure S3 | The UV absorption spectrum for sodium chlorite. The absorption spectrum for the 200-400 nm UV range of 1,000 ppm $NaClO_2$ (Sigma-Aldrich, technical grade 80%) in Nanopure 18.2 MΩ-cm deionized (DI) water with absorption measured on a Cary 60 UV-Vis Spectrophotometer using a quartz cuvette. The absorption peak at 260 nm is from ClO_2^- and matches that for pure (99.98%) $NaClO_2$, while the peak at 292 nm is most likely from the 20% unspecified material and matches the absorption for the OCl^- ion[1-3].

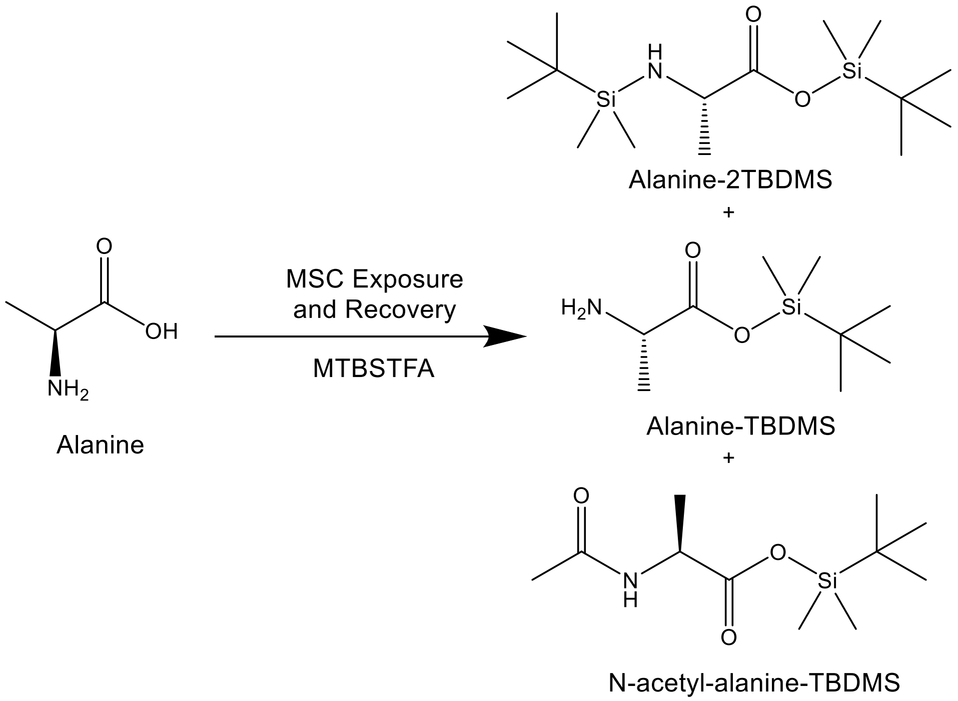

Supplementary Figure S4 | Detection of alteration products using GCMS. Proposed pathway for detection of alanine-2TBDMS along with the alteration products alanine-TBDMS and N-acetyl-alanine-TBDMS. Alanine-coated sand is exposed to humid martian conditions beneath a layer of $NaClO_2$ which evolves to ClO_2 upon exposure to UV radiation. Following exposure, the sand is then leached to extract a matrix of remnant organic products and derivatized with MTBSTFA for analysis and quantification using GCMS. Alanine and resulting evolved products are separated by gas chromatography each peak is then identified by an electron impact ionization mass spectrometer.

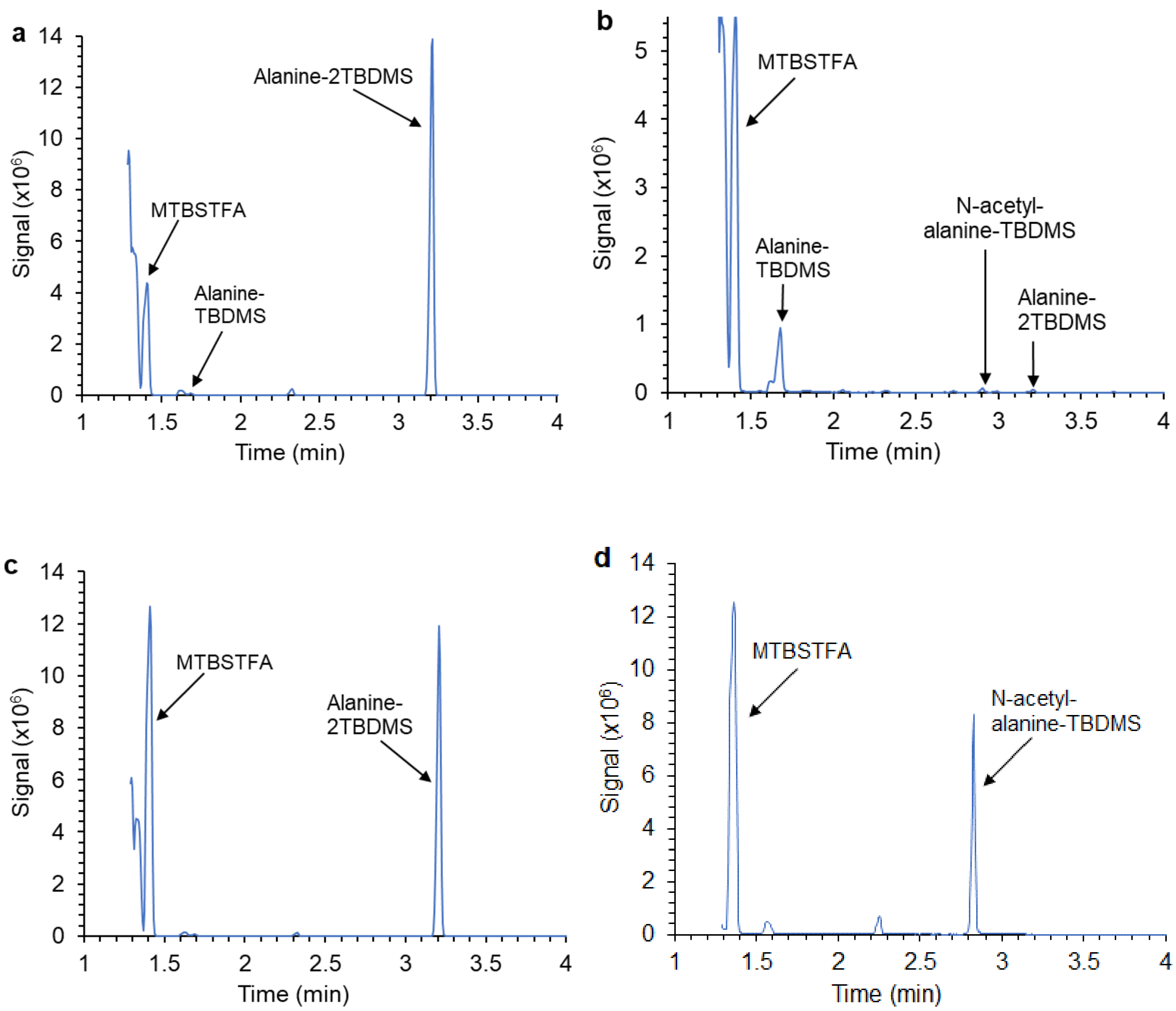

Supplementary Figure S5 | Example chromatographs collected during experiment #2. a, Control alanine-coated sand leachate sample injected into the GCMS following extraction and derivatization. Peaks have been identified as unreacted MTBSTFA, alanine-TBDMS, and alanine-2TBDMS. **b,** Sample exposed in the MSC (Exp. #2) then extracted and derivatized with peaks identified as unreacted MTBSTFA, alanine-TBDMS, N-acetyl-alanine-TBDMS, and alanine-2TBDMS. Following exposure, the alanine-TBDMS peak has increased along with the appearance of the N-acetyl-alanine-TBDMS while the alanine-2TBDMS has significantly decreased in abundance. **c,** Control sample with 250 µL of 1,000 ppm alanine stock solution derivatized with MTBSTFA for comparison with (a) and (b). **d,** Control sample with 250 µL of 1,000 ppm N-acetyl-alanine stock solution derivatized with MTBSTFA for comparison with (b). NOTE: Unlabeled peaks in chromatographs were identified as contaminants and included water, carbonate, and borate, which are susceptible to derivatization but do not otherwise impact the data.

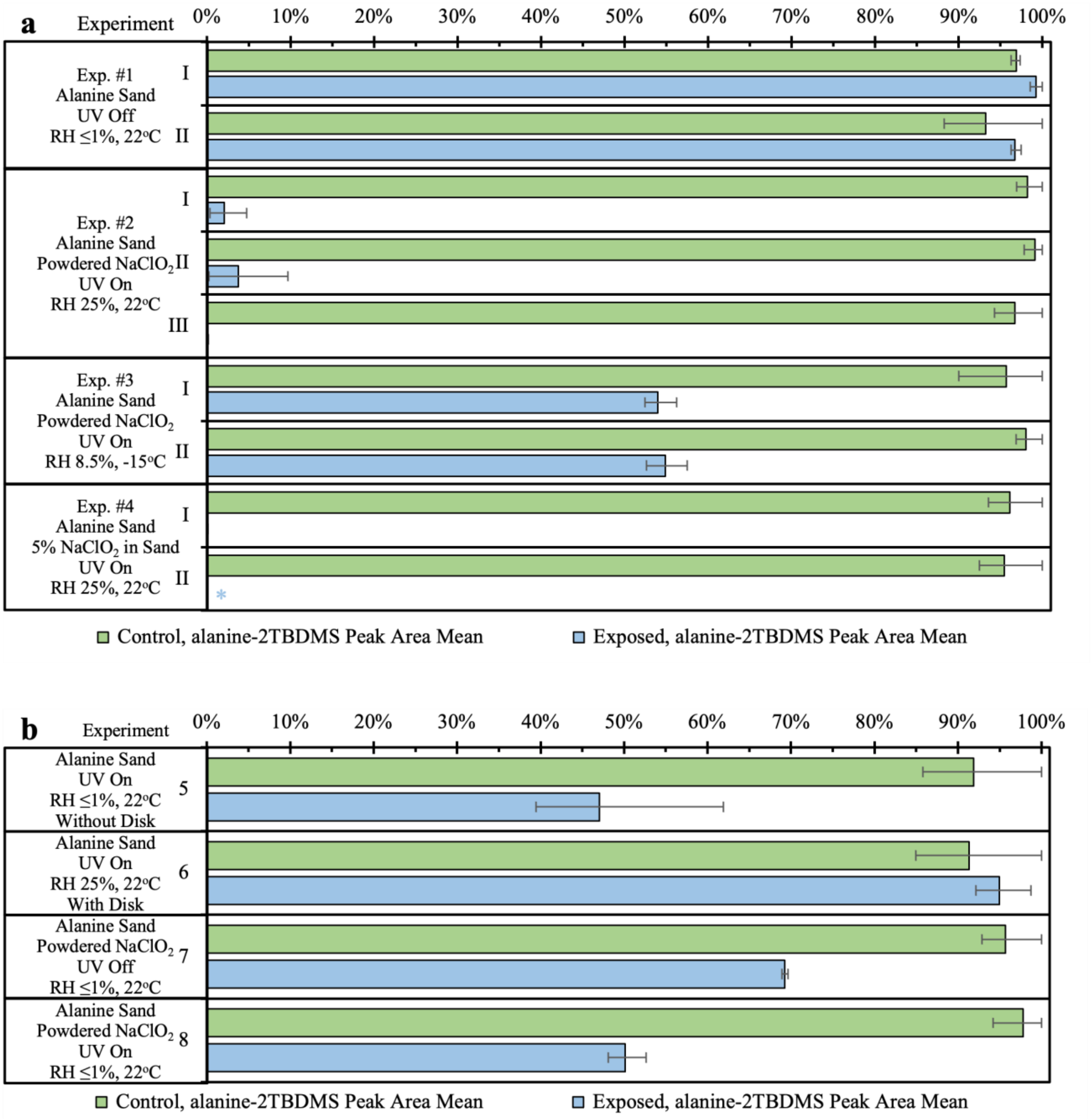

Supplementary Figure S6 | GCMS quantified peak areas of alanine derivatized with MTBSTFA. **a,** Control samples of alanine sand leachate compared with samples exposed to Mars ambient conditions, at ~ 22°C, at -15 ± 0.1°C, with a powdered $NaClO_2$ layer, or a 5% $NaClO_2$ sand layer exposed to UV. Experimental conditions are repeated in duplicate or triplicate for repeatability. **b,** Experimental chamber runs observing the impact of individual factors including UV exposure, microfiber disk coverage of alanine sand, and RH in various combinations. GCMS peak area was used to quantify alanine following derivatization. Detailed values are given in Table S2 * = indicates peak signal was < LOD.

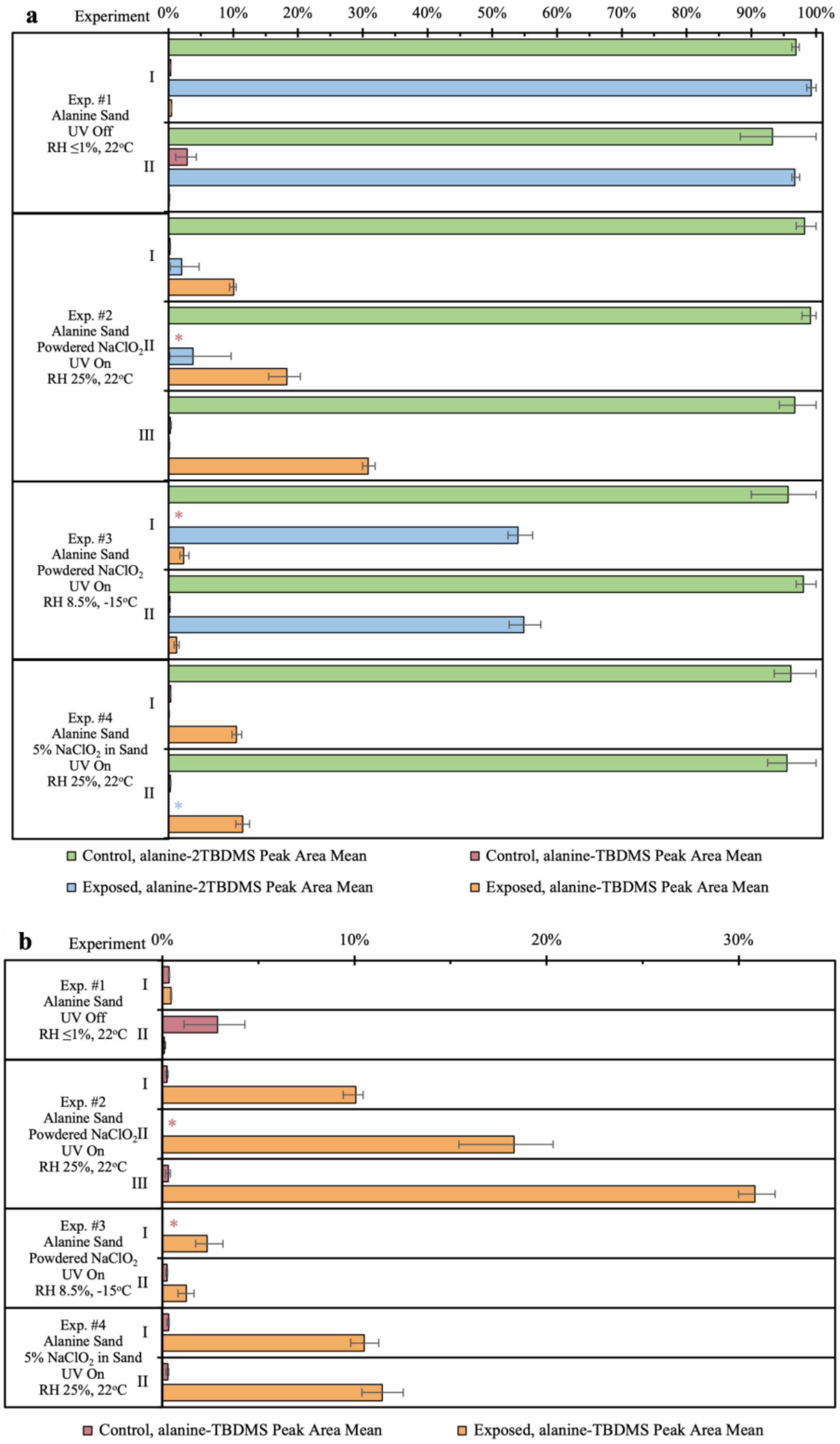

Supplementary Figure S7 | Quantification of alanine-TBDMS. a, GCMS analysis of the sand leachate control and exposed samples from Figure S6a with the addition of alanine-TBDMS peak area quantification normalized to the control alanine-2TBDMS peak area mean. **b,** Quantified alanine-TBDMS peak data only, scaled down to 35% normalization for experimental condition comparison. NOTE: Error bars denote the minimum and maximum peak area for the sample under split injection and are representative of the consistency of the instrumental detection. * = indicates peak signal was <LOD.

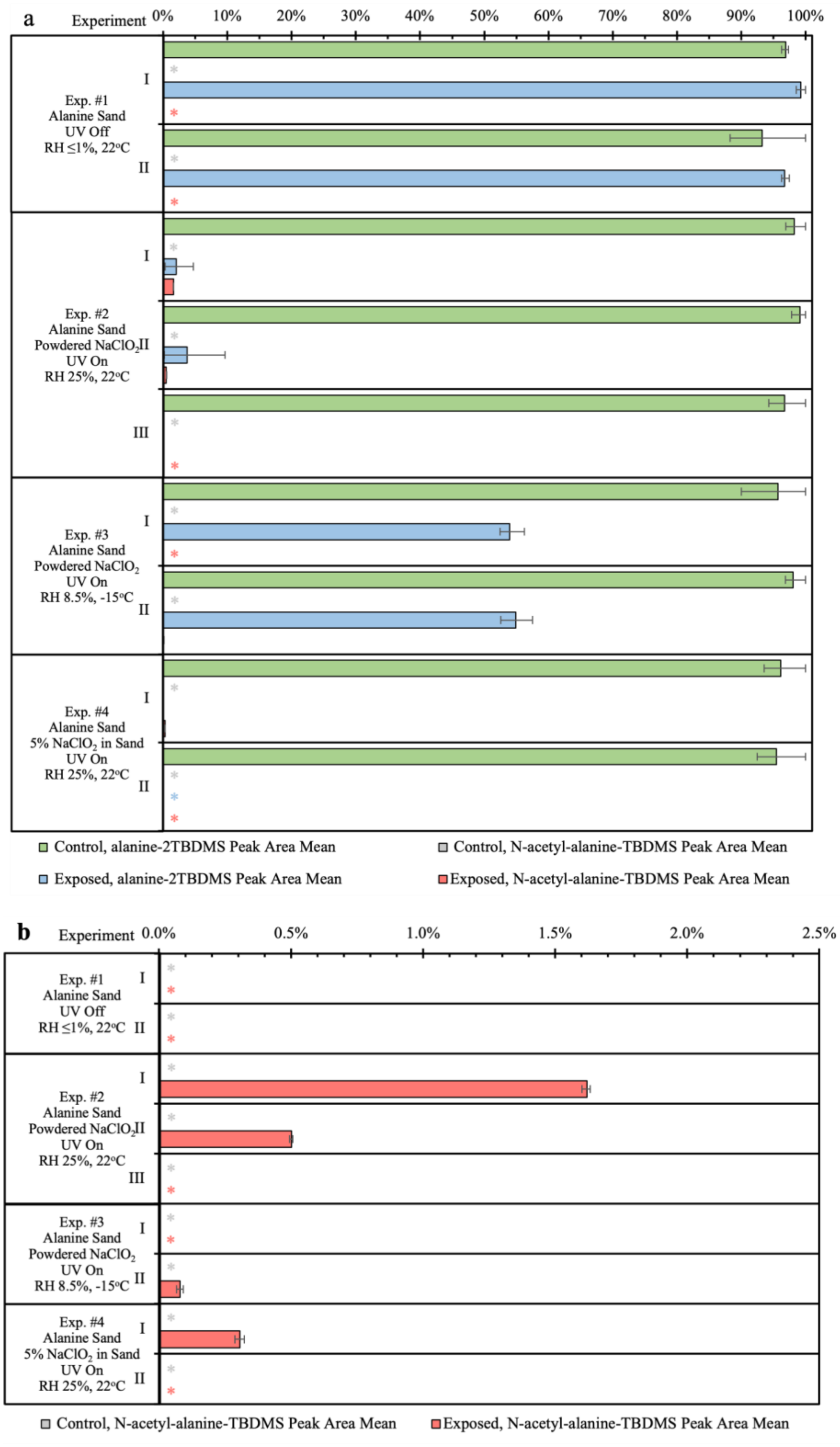

Supplementary Figure S8 | Quantification of N-acetyl-alanine-TBDMS. a, The GCMS analysis of the sand leachate control and exposed samples from Figure S6a with the addition of N-acetyl-alanine-TBDMS peak area normalized to the control alanine-2TBDMS peak area mean. **b,** Quantified N-acetyl-alanine-TBDMS peak data only, scaled down to 2.5% normalization for experimental condition comparison. NOTE: Error bars denote the minimum and maximum peak area for the sample under split injection and are representative of the consistency of the detection. * = indicates peak signal was <LOD.

Experiment 1

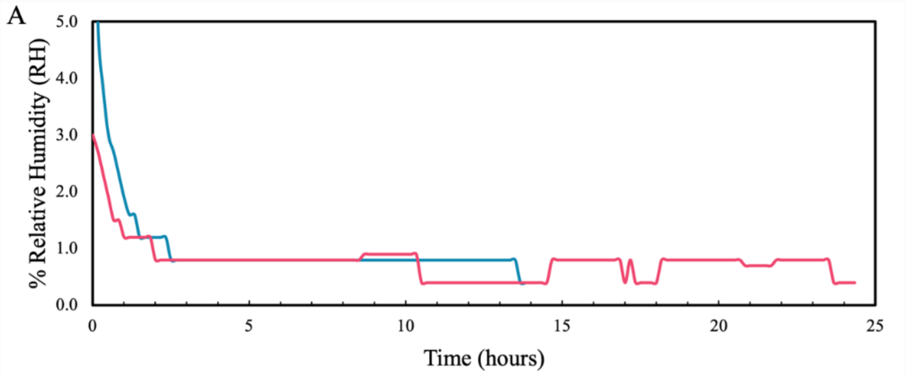

Experiment 2

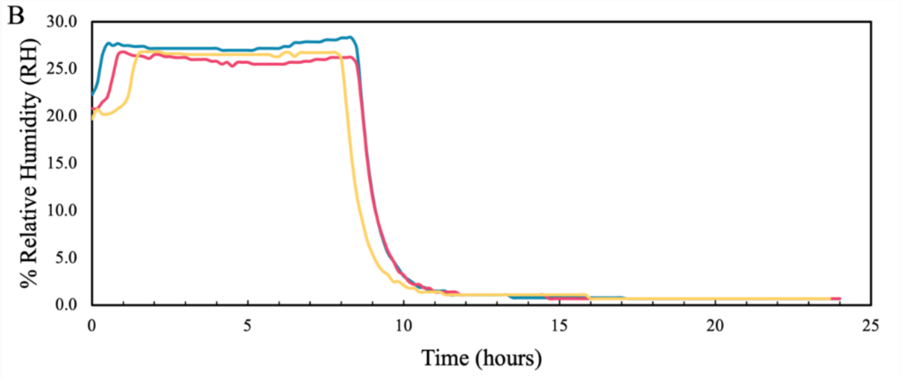

Experiment 3

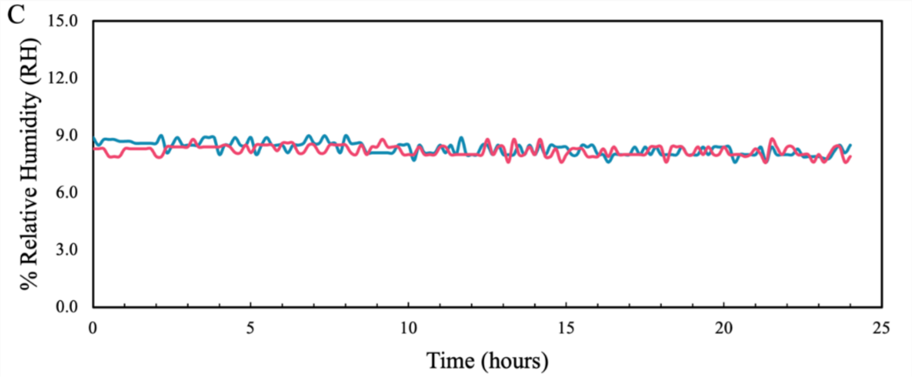

Experiment 4

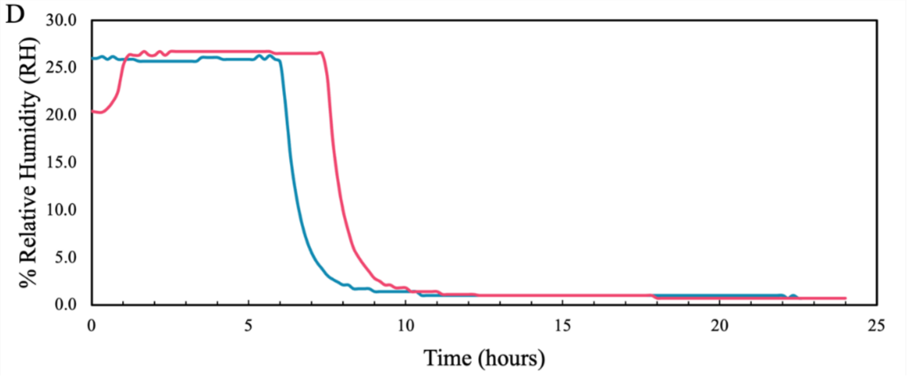

Experiments 5-8

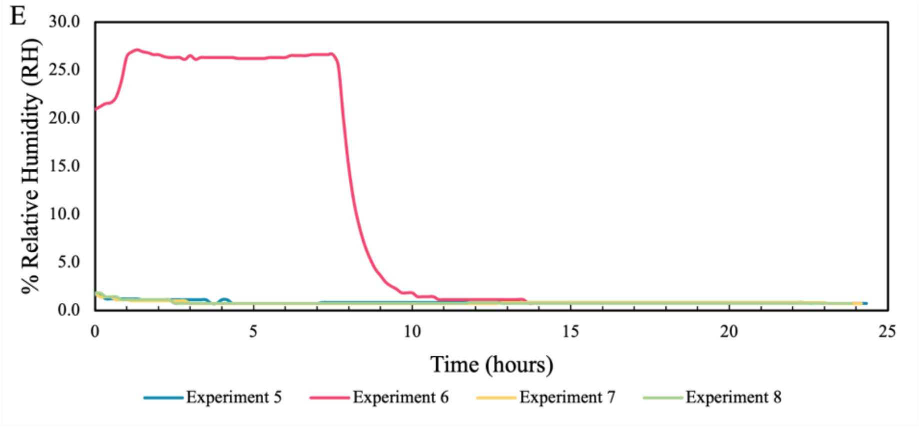

Experiments 5-8 (Scaled to <5% RH)

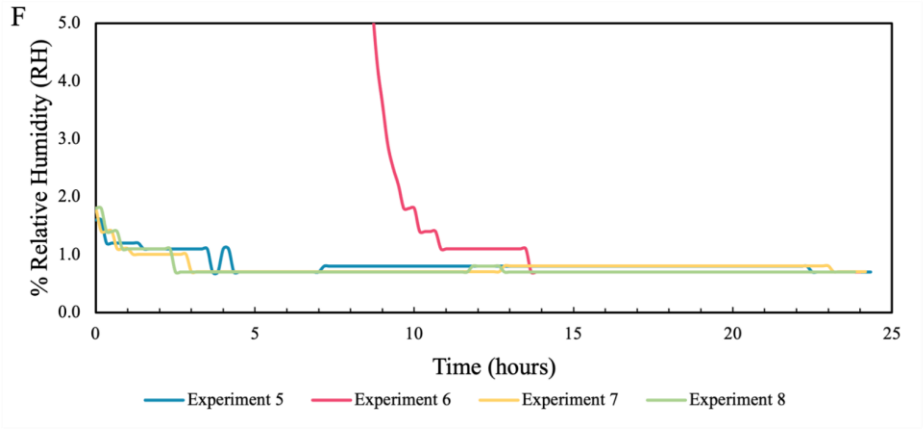

Supplementary Figure S9 | Relative Humidity vs Time for MSC experiments. Data for experiments shown in Fig. S6 clustered into duplicate or triplicates at the same experimental conditions (Table 1) with each color corresponding to an experiment replicate (for a-d). The RH sensor has a resolution of 0.1% and recorded every 10 minutes. **a,** MSC control at ambient temperatures, Mars simulated gas mixture, maintained 7.0 mbar pressure, ~ 22°C, and no additional humidity. **b,** MSC chamber exposure with the addition of 25 g of ice-water at ~ 22°C and UV exposure of powdered $NaClO_2$ layer. **c,** MSC chamber exposure with the addition of 25 g of ice-water and the cold plate set to -15 ± 0.1°C with UV exposure of powdered $NaClO_2$ layer. **d,** MSC chamber exposure with the addition of 25 g of ice-water at ~ 22°C with UV exposure of 5% $NaClO_2$ sand layer. **e,** MSC chamber exposure for various control experiments in Fig. S6b. Exps. #5, #7, and #8 did not consider RH as an experimental factor while Exp. #6 exposed samples to humidity. **f,** Scaled version of Fig. S9e to < 5% RH for ease of interpretation.

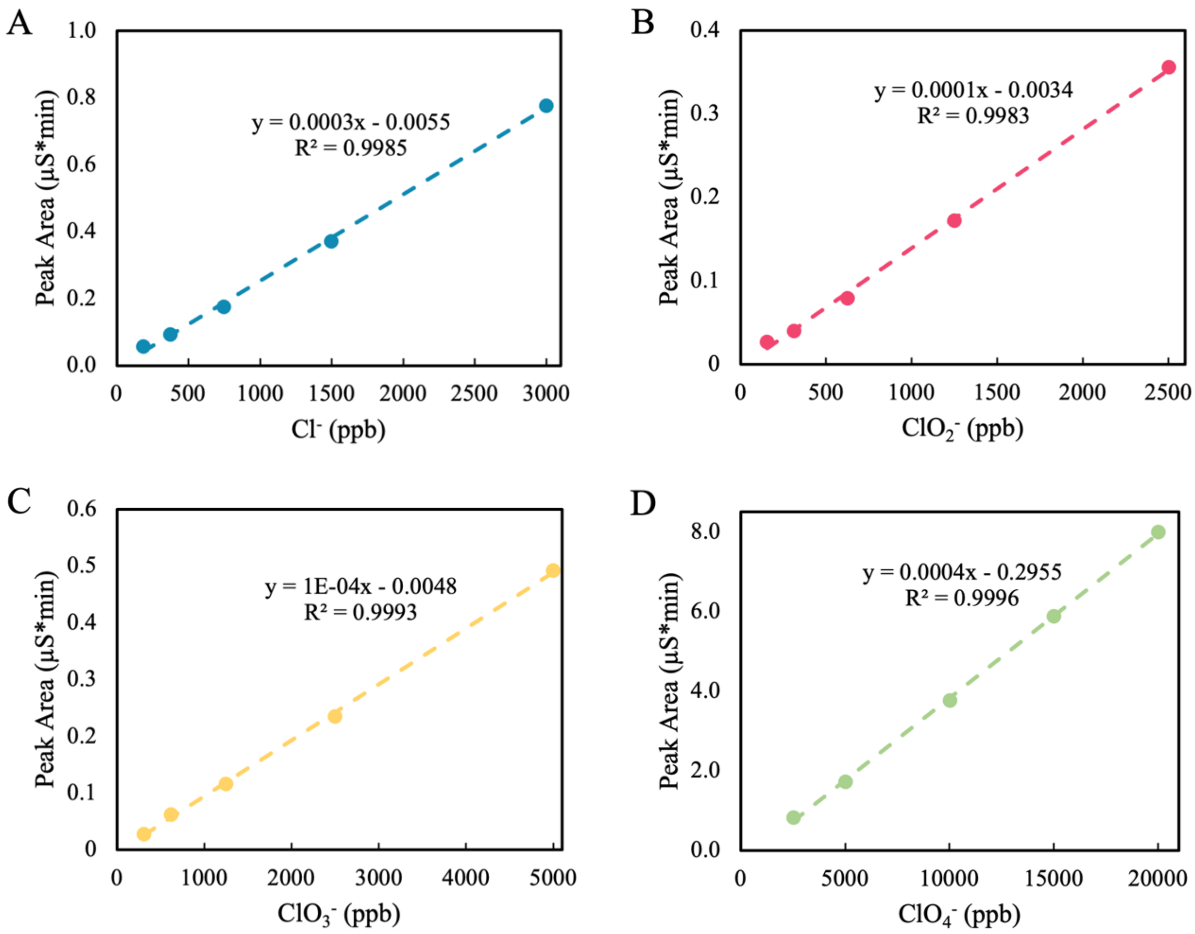

Supplementary Figure S10 | IC calibration curves. Linear relationships and best fit for peak area (µS*min) vs concentration (ppb) used for quantification of IC data in Table S1.

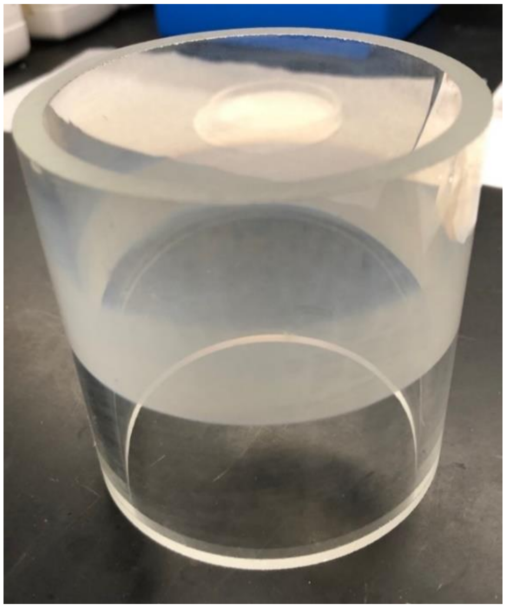

Supplementary Figure S11 | Film on the outer glass cylinder after exposure to gaseous ClO_2. Inner cylinders containing samples were stacked below where the white film appeared, implying that the evolved gaseous ClO_2 not only permeated downwards into the alanine sand mixture but also deposited on the walls of the glass cylinder to form a film. A gradient in the thickness of the film was observed with the thickest film adjacent to the exposed powdered $NaClO_2$ layer.

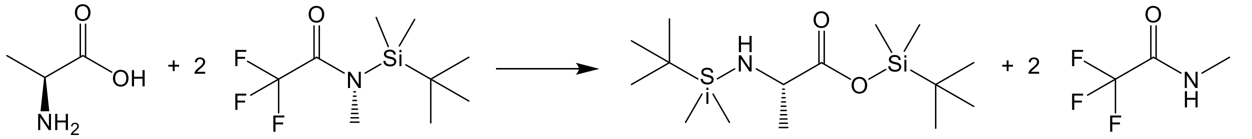

Supplementary Figure S12 | Derivatization pathways. Reaction pathway for the derivatization of alanine with MTBSTFA to form alanine-2TBDMS for GCMS quantification.

Supplementary Tables S1-S3

Supplementary Table S1 | Analysis of oxychlorine ions. Chloride and oxychlorine ions recovered in the leachate from the alanine-coated sand layer beneath a layer of powdered $NaClO_2$ and isolated by the glass microfiber disk. Humid MSC exposure at ~ 22°C (ambient) as well as with the internal cold plate set to -15 ± 0.1°C (under conditions A1 and A2). Chloride and oxychlorine ions were recovered from the internal walls of the permeation chamber glass cylinder by swabbing with the tip dipped into DI water. Chamber exposure remained constant at ambient temperatures for all experiments, while the mini-MSC was sealed with recorded internal RHs (under conditions B1-B3). All controls were < LOD for all ions. LOD = Limit of Detection. LOQ = Limit of Quantification.

Ion	**17 g Alanine-Coated Sand** (µmol)		**Swab of Thin Film on Cylinder Wall** (µmol)		
	**(A1)** 25% RH, ~ 22ºC	**(A2)** 8.5% RH, -15 ± 0.1ºC	**(B1)** 8.3% RH ~ 22ºC	**(B2)** 16.2% RH ~ 22ºC	**(B3)** 28.5% RH ~ 22ºC
**Cl^-**	1.80 ± 0.02	1.09 ± 0.01	< LOQ	1.10 ± 0.06	24.6 ± 0.1
**ClO_2^-**	< LOQ	0.75 ± 0.01	< LOD	< LOD	1.78 ± 0.02
**ClO_3^-**	3.20 ± 0.04	0.40 ± 0.02	< LOD	0.41 ± 0.01	38.4 ± 0.1
**ClO_4^-**	< LOQ	< LOD	58.9 ± 0.2	41.0 ± 0.1	79.3 ± 0.1

Supplementary Table S2 | Determination of derivatized alanine. Quantitative integrated peak area from GCMS analysis of derivatized alanine with alanine-coated sand samples placed in the MSC and normalized with the alanine sand control. Experimental data for alanine-2TBDMS, alanine-TBDMS, and N-acetyl-alanine-TBDMS following exposure to various specified conditions in the MSC. **a,** Numerical data plotted in Figure 3 and Figures S6, S7, and S8. **b,** Numerical data for alanine-TBDMS and N-acetyl-alanine-TBDMS for data plotted in Figure 3 and Figure S6. LOD = Limit of Detection. LOQ = Limit of Quantification.

**a** Experimental specifications in the MSC and experiment number	Normalized % Peak Area for alanine-2TBDMS		Normalized % Peak Area for alanine-TBDMS		Normalized % Peak Area for N-acetyl-alanine-TBDMS	
	Control	Exposed	Control	Exposed	Control	Exposed
**#1 -** Alanine Sand, MSC Control, n = 2	95 ± 2	98 ±1	< LOD	< LOD	< LOD	< LOD
**#2** - Alanine Sand, Powdered $NaClO_2$, UV, ~ 25% RH, n = 3	98 ± 1	2 ± 1	< LOD	20 ± 9	< LOD	1.5 ± 1
**#3** - Alanine Sand, Powdered $NaClO_2$, UV, ~ 8.5% RH, -15 ± 0.1°C Cold Plate, n = 2	97 ± 1	54 ± 1	< LOD	2 ± 1	< LOD	< LOQ
**#4** - Alanine Sand, 5% $NaClO_2$ in Sand, UV, ~ 25% RH, n = 2	96 ± 1	< LOD	< LOD	11 ± 1	< LOD	< LOQ

**b** Experimental specifications in the MSC and experiment number	Normalized % Peak Area for alanine-2TBDMS		Normalized % Peak Area for alanine-TBDMS		Normalized % Peak Area for N-acetyl-alanine-TBDMS	
	Control	Exposed	Control	Exposed	Control	Exposed
**#5** - Alanine Sand, No Disk Cover, UV Exposure	92 ± 2	47 ± 2	< LOD	< LOD	< LOD	< LOD
**#6** - Alanine Sand, No $NaClO_2$, UV, ~ 25% RH	91 ± 2	95 ± 2	< LOD	< LOD	< LOD	< LOD
**#7** - Alanine Sand, Powdered $NaClO_2$, No UV, No RH	96 ± 2	69 ± 2	< LOD	< LOD	< LOD	< LOD
**#8** - Alanine Sand, Powdered $NaClO_2$, UV, No RH	98 ± 2	50 ± 2	< LOD	< LOD	< LOD	< LOQ

Supplementary Table S3 | Analysis of $NaClO_2$ for ClO_x^- after UV exposure. Commercial sodium chlorite ($NaClO_2$) is compared with $NaClO_2$ exposed to UV irradiation and 16.2% RH and ~ 22°C isolated in the mini-MSC experiment for film generation and recovery in Table S1. Stock solutions of 1,000 ppm $NaClO_2$ in DI water before and after exposure were diluted as needed and analyzed by IC. Exposure increased the concentration of Cl^-, produced ClO_3^- not present before exposure, and resulted in a decrease of ClO_2^- in exposed material. There was no ClO_4^- detected in any samples. *Some values were obtained by extrapolating slightly beyond the calibration curve, but note that R^2 = 0.9993. LOD = Limit of Detection.

Ion	Control	Following MSC Exposure to UV at 16.2% RH
	ppb in solution	ppb in solution
Cl^-	*264 ± 13	425 ± 21
ClO_2^-	889 ± 44	434 ± 23
ClO_3^-	*38 ± 2	329 ± 16
ClO_4^-	<LOD	<LOD.

Supplementary References

1 Furman, C. S. & Margerum, D. W. Mechanism of Chlorine Dioxide and Chlorate Ion Formation from the Reaction of Hypobromous Acid and Chlorite Ion. *Inorg. Chem*. **37**, 4321-4327 (1998).

2 Kishimoto, A. et al. Elucidation of composition of chlorine compounds in acidic sodium chlorite solution using ion chromatography. *PLoS One* **18** (2023).

3 Philippi, M., dos Santos, H. S., Martins, A. O., Azevedo, C. M. N. & Pires, M. Alternative spectrophotometric method for standardization of chlorite aqueous solutions. *Anal. Chim. Acta* **585**, 361-365 (2007).
